# Muscles that move the retina augment compound-eye vision in *Drosophila*

**DOI:** 10.1038/s41586-022-05317-5

**Published:** 2022-10-26

**Authors:** Lisa M. Fenk, Sofia C. Avritzer, Jazz L. Weisman, Aditya Nair, Lucas Randt, Thomas L. Mohren, Igor Siwanowicz, Gaby Maimon

**Affiliations:** 1 Laboratory of Integrative Brain Function and Howard Hughes Medical Institute, The Rockefeller University, New York, New York, USA.; 2 Active Sensing, Max Planck Institute for Biological Intelligence (in foundation), Martinsried, Germany; 3 Present address: Department of Biology, Massachusetts Institute of Technology, Cambridge, Massachusetts, USA.; 4 Janelia Research Campus, Howard Hughes Medical Institute, Ashburn, Virginia, USA.

## Abstract

The majority of animals have compound eyes, with tens to thousands of lenses attached rigidly to the exoskeleton. A natural assumption is that all these species must resort to moving either their head or body to actively change their visual input. However, classic anatomy has revealed that flies have muscles poised to move their retinas under the stable lenses of each compound eye^1–3^. Here we show that *Drosophila* use their retinal muscles to both smoothly track visual motion, which helps to stabilize the retinal image, and also to perform small saccades when viewing a stationary scene. We show that when the retina moves, visual receptive fields shift accordingly and that even the smallest retinal saccades activate visual neurons. Using a new head-fixed behavioral paradigm we find that *Drosophila* perform binocular, vergence movements of their retinas—which could enhance depth perception—when crossing gaps and impairing retinal-motor-neuron physiology alters gap-crossing trajectories during free behavior. That flies evolved an ability to actuate their retinas argues that moving the eye independently of the head is broadly paramount for animals. The similarities of smooth and saccadic movements of the *Drosophila* retina and the vertebrate eye highlights a notable example of convergent evolution.

Eye movements serve many perceptual, cognitive, and social functions for primates and other species with single-lens eyes. Most animals, however, have compound eyes that attach rigidly to their heads. *Drosophila melanogaster*, for example, have compound eyes and this species serves as an important model for understanding the neural basis of vision. Might *Drosophila*, somehow, actively move their eyes even when their head is still? If so, what functions might such eye movements serve and how might their dynamics compare to eye-movement dynamics in primates and other vertebrates?

In the 1970s, it was discovered that houseflies have a muscle under each compound eye that attaches to the *orbital ridge*, a thin sheet of cuticle that surrounds the retina and part of the optic lobes^1,2^. This muscle seemed poised to shift the optical axes of the photoreceptors relative to the lenses of the compound eye^1,2,4^. “Clock spikes”—large, extracellularly recorded action potentials with regular inter-spike intervals evident in the vicinity of the optic lobes—were shown to reflect the activity of a motor neuron innervating this retinal muscle^2^. In 1991, a second muscle in houseflies was discovered, suggesting that the retina might move in two dimensions^3^. The rate of clock spikes is modulated spontaneously and also in response to external stimuli^4–7^. In preliminary reports, Franceschini and colleagues have argued that these spike-rate modulations correlate with movements of photoreceptors^3,8,9^. In *Drosophila*, recent work has focused on phototransduction-based movements of photoreceptors^10–12^ and thus despite the initial housefly observations, the functions of muscular movements of the fly retina^13^ remain unclear.

## Two muscles move the *Drosophila* retina

**Figure 1.**
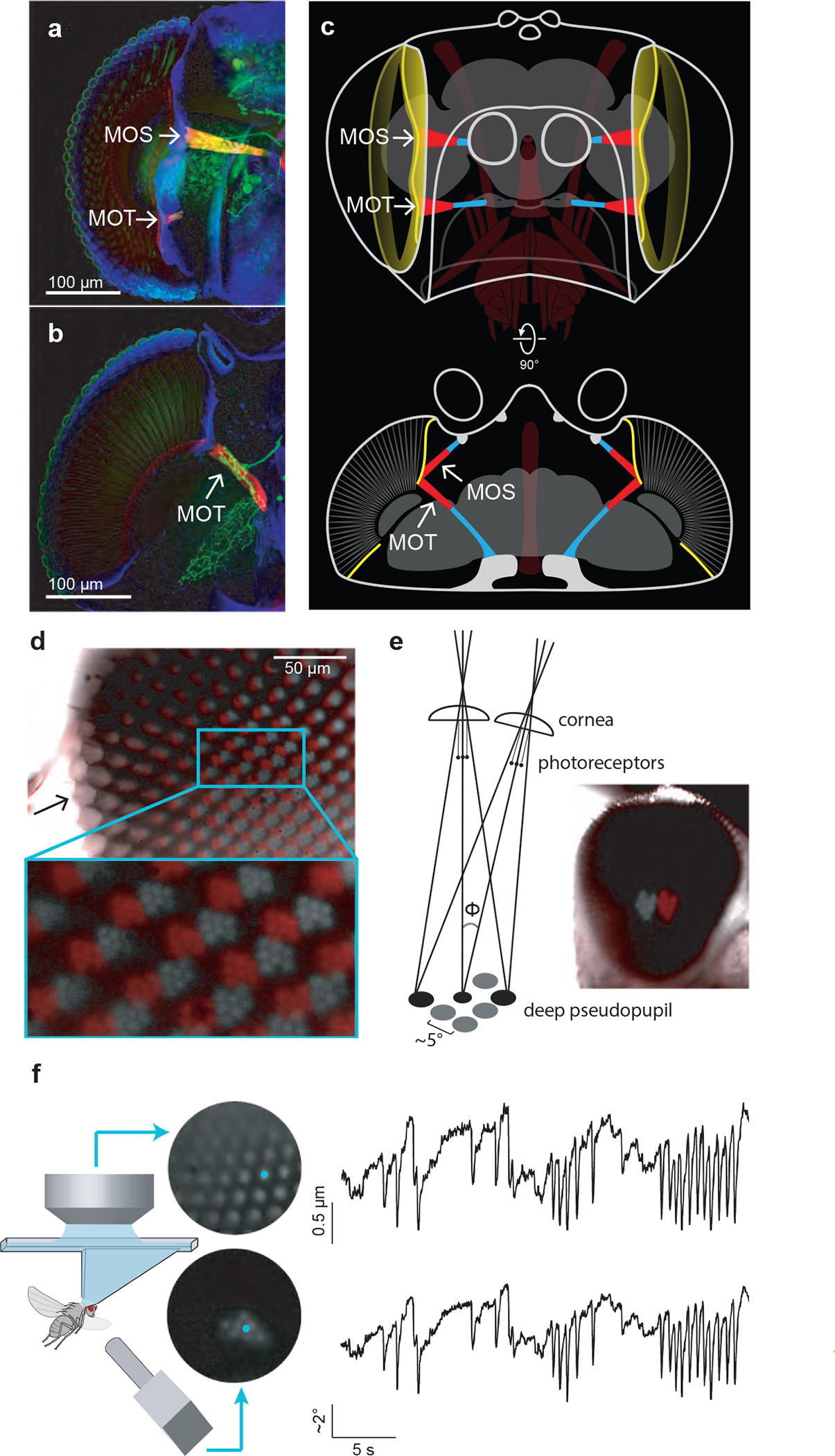
*Drosophila* have two muscles per eye which act to move the retina. **(a)** Frontal view through the *Drosophila* head showing immunostaining for muscles in red (phalloidin) and neurons, including retinal motor neurons, in green (mVenus). *Musculus orbito-scapalis* (MOS), which interconnects the antennal cup to the front of the orbital ridge (which surrounds the retina), is fully visible. *Musculus orbito-tentoralis* (MOT) is partially visible, at its insertion point to the orbital ridge. **(b)** A horizontal view allows one to fully visualize the MOT and its innervating motor neuron (arrow). **(c)** Corresponding schematics of the insertion of both muscles in the coronal (top) and horizontal (bottom) plane. Muscle tendons in blue, orbital ridge in yellow. Other muscles (proboscis, esophagus) in brown. **(d)** Retina seen through a water immersion objective. Two images are overlaid. The red image shows the retina during optogenetic activation of the retinal motor neurons, yielding a maximal shift of photoreceptor tips. The grey image was taken after turning off the optogenetic light, with the muscles fully relaxed. Note that whereas the photoreceptors moved with optogenetic activation (red and gray photoreceptor tips are offset), the lenses, which are in focus on the left of the image (arrow), were stable. **(e)** The schematic shows how the deep pseudopupil^16^ is an erect, virtual image of the photoreceptor tips at the center of the curvature of the compound eye (drawn after^16^). Overlayed image of the deep pseudopupil in one fly during (red) and after (gray) optogenetic activation (as in panel d). **(f)** Simultaneously tracked center-of-mass traces of photoreceptor tips, via a water immersion objective visualizing ommatidia at the very top of the eye, and the deep pseudopupil, via an air lens aimed at a lower region of the same eye. See methods for how we converted pseudopupil movements from the units of pixels to degrees.

We discovered that *Drosophila*, like houseflies, have two retinal muscles (Fig. 1a–c, Extended Data Movies 1,3). One muscle resembles the *musculus orbito-tentoralis* of large flies^1–3^. The muscle is attached via a long tendon to the tentorial bar posteriorly and inserts into the antero-medial rim of the orbital ridge. A second muscle, which resembles the *musculus orbito-scapalis*^3^ of large flies, inserts more dorsally, into the frontal-medial rim of the orbital ridge. This second muscle originates at the edge of the antennal cup, to which it connects via a short tendon.

Because these muscles seem poised to move the *Drosophila* retina, we visualized the tips of the photoreceptors with a water-immersion objective positioned above the eye (Methods)^14^. We expressed the red-shifted channelrhodopsin, CsChrimson^15^, in the motor neurons innervating the retinal muscles. Via a pulse of red light, we optogenetically drove the retina to an extreme, tensed position (Fig. 1d, red) and then let it relax to an extreme, relaxed position by turning off the light (Fig. 1d, gray). We observed peak-to-peak displacements of the photoreceptors that spanned ~3 inter-photoreceptor spacings, which corresponds to ~15° in angular space (Fig. 1d).

Submerging the fly eye in water precludes many experiments. We thus decided to track the photoreceptor tips by measuring the position of the *deep pseudopupil* ^16^—an enlarged, virtual image of photoreceptors that forms at the center of curvature of the compound eye, which can be visualized with an air lens (Fig. 1e). Specifically, when one places a point source of light abutting the fly, such that light exiting the compound-eye is collected by the air lens, the deep pseudopupil appears as seven large, bright dots, arranged in the shape of a single ommatidium’s receptor array. We used 780 or 850 nm light—wavelengths to which the fly’s natural opsins are insensitive^17^—to visualize the deep pseudopupil without blinding the fly. To verify that movements of the deep pseudopupil accurately reflect photoreceptor movements, we simultaneously tracked the deep pseudopupil (with an air lens) and photoreceptors at the top of the eye (with a water-immersion objective) and observed a tight correlation between these two signals (Fig. 1f, Extended Data Movie 4). We observed the same maximal angular excursion of ~15° in the deep pseudopupil during optogenetic activation of the retinal muscles as when tracking the positions of the photoreceptors directly (Fig. 1d–e).

## Retinal movements shift receptive fields

**Figure 2.**
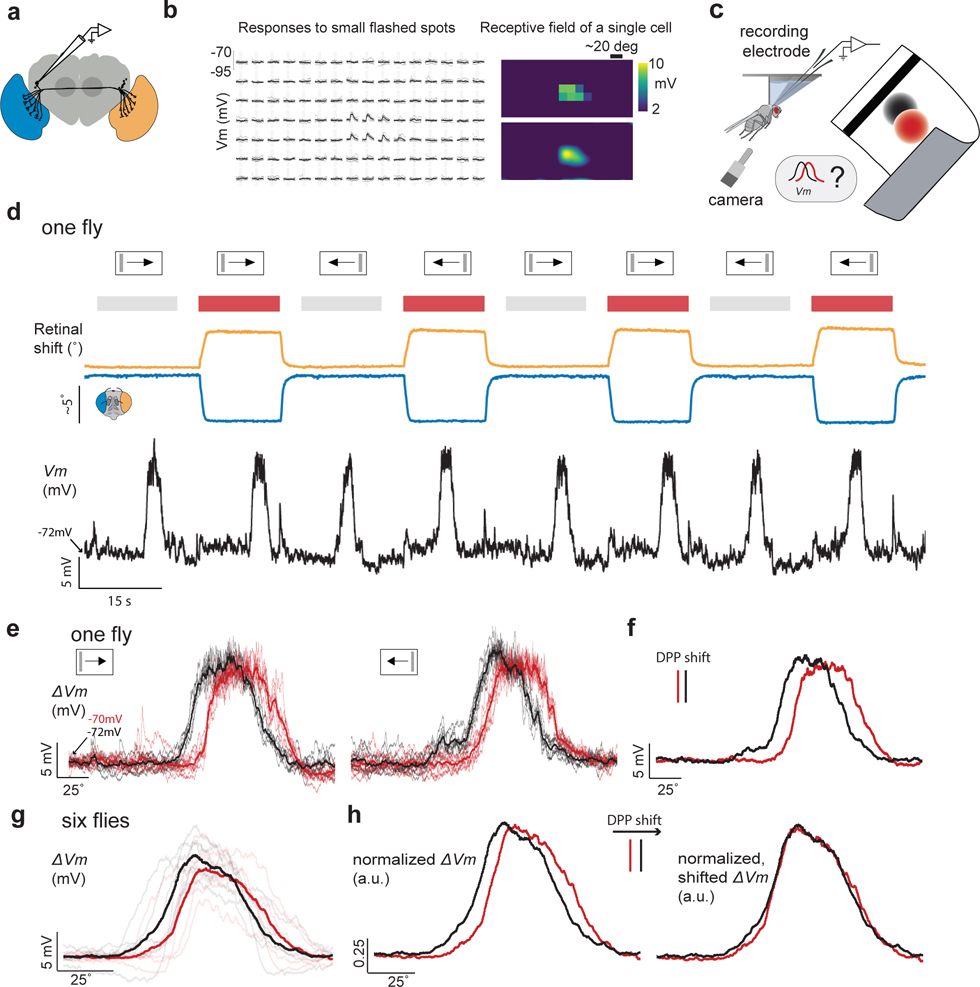
Retinal movements yield the expected angular shifts in receptive fields of visual neurons. **(a)** We recorded from LC14 neurons on the right side. **(b)** Example of an LC14 cell 2D receptive field, as estimated by responses to 100 ms (gray region) flashes on the screen. **(c)** We performed whole cell patch clamp recordings in rigidly tethered flies. We optogenetically activated the motoneurons (Methods) to induce large retinal shifts. We measured the concomitant retinal position with a camera as well as the membrane voltage (*Vm*) in visual neurons in response to a moving black bar (bar width: 9 deg width, bar velocity: 21 deg/s) in lieu of the 2-dimensional receptive field. **(d)** Example trace showing the retinal positions (top trace; orange: left retina: right retina) and the *Vm* (bottom). Grey and red rectangles indicate trials without and with optogenetic activation. **(e)** Left: Baseline subtracted membrane voltage for rightward bar motion in one fly. Red thin lines show trials with optogenetic activation (n = 9) and black lines trials without (n = 7), thick lines indicate the means. Right: As left, but for leftward bar motion (n = 9 for both conditions). **(f)** Averaged membrane voltage across trials and for both bar directions. Distance between red and black vertical lines indicates the shift in the pseudopupil induced by the optogenetic light. **(g)** As in **(f)** but for a population of six flies (light lines single flies, dark lines averages). Baseline *Vm* (mean during 0.5 s at trial onset) was in all flies slightly higher during optogenetic activation than in trials without (mean *Vm*: −71.1 vs −72.5 mV with standard deviations of 5.5 mV). **(h)** Left: As in **(g)** but for single fly data normalized to peak values. Right: *Vm* data is shifted by the measured angular retinal shift (indicated by vertical lines).

*Drosophila* and other dipterans have *neural superposition* eyes, where each of the eight photoreceptors in a single ommatidium is in precise optical alignment to specific photoreceptors in neighboring ommatidia. Signals from all photoreceptors oriented along a common angle in visual space—independently of their ommatidium of origin—are ultimately combined in downstream neurons via a sophisticated neural wiring scheme^18^. If retinal muscles were to move the optical axes of photoreceptors in different ommatidia by different amounts, this would compromise the fidelity of neural superposition and thus impair the ability of visual neurons to signal effectively. To address this concern, we measured the visual responses of LC14 cells^19^ (or *dorsal cluster neurons*^20^) via whole-cell patch-clamp recordings during optogenetically induced retinal movements. LC14 cells interconnect the two visual lobes^19,20^. We found that they have ~50° wide receptive fields along the frontal, vertical midline (Fig. 2a–c). These cells respond strongly to moving bars or spots traversing their receptive fields (Extended Data Fig. 2).

Using a vertical bar that swept left or right in front of the fly, we measured the horizontal (yaw) position of LC14 receptive fields in the context of different retinal positions. To achieve two stable retinal positions, we either bilaterally activated retinal motoneurons optogenetically, or left them unactivated, as the bar swept. The example trace in Figure 2d shows consistent, depolarizing membrane voltage (*Vm*) responses to a bar moving across the receptive field, independently of whether the retina was optogenetically repositioned or not. Importantly, however, the trial-averaged *Vm* traces revealed that this LC14 cell responded to the bar at slightly different positions on the screen during optogenetic activation compared to control trials (Fig. 2e, red versus black). Because LC14 cells are insensitive to the direction of visual motion (Extended Data Fig. 2), we could combine trials in which the bar moved to the left and to the right to yield the best possible estimate of the shift in the cell’s receptive field with optogenetic activation (Fig. 2f). All six LC14 cells that we recorded showed a receptive-field shift in the expected direction. Moreover, when we slid the population-averaged *Vm* curves with and without optogenetic activation by the mean pseudopupil (i.e. retinal image) displacement (5.7°), we observed a precise match of the *Vm* curves at the two retinal positions (Fig. 2g,h). The fact that retinal movements lead to precise shifts in the position of LC14 receptive fields, rather than degrading their visual responses more generally, argues that effective neural superposition is preserved at varied retinal positions.

## Visually induced retinal movements

**Figure 3.**
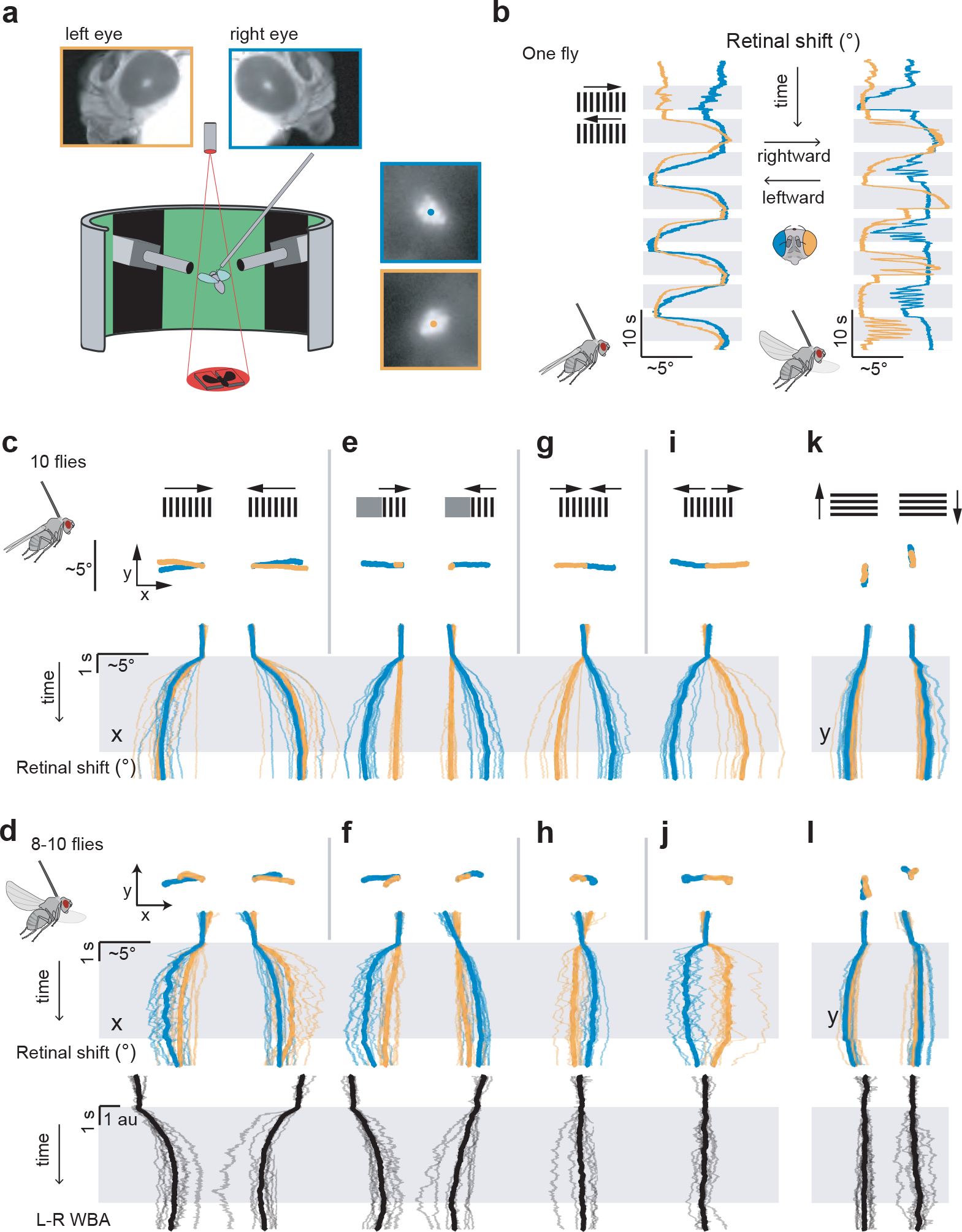
*Drosophila* perform vertical and horizontal retinal optokinetic responses and these responses are independently controlled in the two eyes. **(a)** Apparatus to monitor the position of the deep pseudopupil in both eyes alongside the wing steering behavior of tethered, flying flies, while visual stimuli are presented on a panoramic LED display. Left eye: orange throughout. Right eye: blue throughout. **(b)** Sample traces of the positions of both pseudopupils as a fly viewed rightward and leftward moving gratings. Data from a quiescent fly is shown on left and a flying fly on right. Responses in flight are more variable, with varying frequency of nystagmus saccades on a trial to trial basis (compare left and right eye in flight). **(c)** Plots at top show the population-averaged 2-dimensional (x-y) movements of the pseudopupils in response to full-field rightward and leftward moving gratings. Traces in middle show the horizontal (x) component of the population-averaged pseudopupil movements over time (Gray region indicates when the stimulus is moving). Schematics on top illustrate the visual stimulus presented. **(d)** Data from the same flies as in c, but during flight. Wing-steering left-minus-right wingbeat amplitude (L–R WBA) responses are shown at bottom, with rightward deflections indicating a rightward steering response. **(e-f)** Same as panels c-d, but for unilateral rightward and leftward motion. Data from gratings presented to the fly’s right eye are shown; symmetrical results were obtained when the left eye was stimulated (not shown). **(g-h)** Same as panels c-d, but for bilateral back-to-front motion. (i-j) Same as panels c-d, but for bilateral front-to-back motion. **(k-l)** Same as panels c-d, but for up and down motion. Vertical (y) movements are shown in the pseudopupil time series for these panels. Thin lines: single fly averages (3–10 trials). Thick lines: population average (N=8–10 flies).

Many animals move their eyes to help stabilize gaze^21^. We wondered whether retinal movements might serve this function in flies. This would be a role akin to the optokinetic reflex in humans, where our eyes smoothly move in the direction of a rotating panoramic scene, which acts to minimize visual motion on our retinas. We tethered flies to tungsten pins with their heads rigidly glued to their thorax and placed them at the center of a panoramic LED display (Fig. 3a). We tracked the position of the pseudopupil of each eye independently. In some experiments, we had the flies perform tethered flight, in which case we simultaneously measured their wing-steering behavior (Methods).

When we rotated a panoramic squarewave grating around a quiescent, non-flying fly (Fig. 3b, left), we observed smooth, direction-selective, tracking movements of the retina in both eyes. Specifically, when the grating moved to the right, the retina moved smoothly to the left and vice versa. In flight, the same fly showed smooth tracking as well but with interspersed resetting movements in the other direction, i.e., counter-saccades (Fig. 3b, right), akin to nystagmus saccades in human optokinetic responses. Responses in flight were more variable, with varying frequency of nystagmus saccades on a trial-to-trial basis (compare left and right eye in flight). This fly’s retinal movements were characteristic of the average movements in our population (Fig. 3c,d) (Extended Data Movies 5 and 6). Counter-saccades occur at unpredictable times and thus are not easily evident in the population-averaged responses (Extended Data Fig. 3). We note that in flight and in quiescence, we observed a tight correlation between the peak velocity of saccades and their amplitudes (Extended Data Fig. 4a,b), consistent with a power law relationship, reminiscent of the ‘main sequence’ in human saccades^22^.

The direction in which the fly retina moves in response to visual motion is opposite to the direction in which the lens of our own eye moves in response to the same visual stimulus, but both movements have the same slowing effect on visual motion. The key point is that when the human eye rotates, both the lens and retina move together whereas in *Drosophila* only the retina moves while the overlying lenses remain stationary. Because the lenses of the fly (and human) eye form inverted images, the fly retina, moving alone, has to move in a direction opposite to the direction of visual motion in order to slow down the movement of the image.

Unlike in humans, the optokinetic reflex in *Drosophila* can operate independently in each eye. When we presented a grating to the right eye only, the right retina tracked while the left retina remained stationary (Fig. 3e,f, Extended Data Movie 7) and vice versa (data not shown). Similarly, when we presented back-to-front motion to both eyes, this elicited front-to-back movements of both retinas (i.e. divergence) (Fig. 3g,h) and when we presented front-to-back visual motion to both eyes, both retinas moved back-to-front (convergence) (Fig. 3i,j).

The fact that *Drosophila* have two retinal muscles per eye (Fig. 1) suggests that they can move their retinas in two dimensions. Indeed, flies also performed a vertical optokinetic response to up and down visual motion (Fig. 3k,l) with peak-to-peak magnitudes that were about 50% the size of horizontal movements.

The mean initial retinal speed in response to gratings moving horizontally at 15°/s was ~3°/s (Extended Data Fig. 5a–b) and thus too slow to fully cancel the visual motion experienced by the retina. This low gain is consistent with the values observed in mice^23^ and goldfish^24^, but not with those observed in primates, where optokinetic gains can approach one^25,26^. It is unlikely that this incomplete cancellation of visual motion is due to muscular constraints, since the retina can move an order of magnitude faster during counter-saccades, with peak velocities exceeding 140°/s (Extended Data Fig. 4) and we also observed spontaneous saccades (described later) that exceeded 600°/s. In unrestrained animals, retinal movements likely work in synergy with head and body movements ^27–33^ to stabilize the visual image and thus they may not need to operate with a very high gain.

Recently it was argued that *photocontraction*^34^—photoreceptor movements resulting from the subcellular mechanics of rhabdomeric phototransduction—can induce *Drosophila* photoreceptors to physically move *in vivo*^10^. If the retinal movements we observed were due to photocontraction, they should persist in flies with intact photoreceptors but impaired downstream visual processing. We silenced the synaptic output of the major lamina visual neurons (L1-L4)^35,36^, which are monosynaptically downstream of photoreceptors, by expressing active tetanus toxin light chain in those cells^37^. Optokinetic responses to visual motion stimuli were entirely abolished after this manipulation (Extended Data Fig. 6), whereas control flies expressing an inactive form of the toxin showed robust retinal movements. These data alongside the bidirectionality of the optokinetic response—which requires the post-photoreceptor calculation of the direction of visual motion—and other results (Extended Data Fig. 7, Supplementary Text), strongly argue that the retinal movements we describe are not due to photocontraction.

## Spontaneous retinal movements

**Figure 4:**
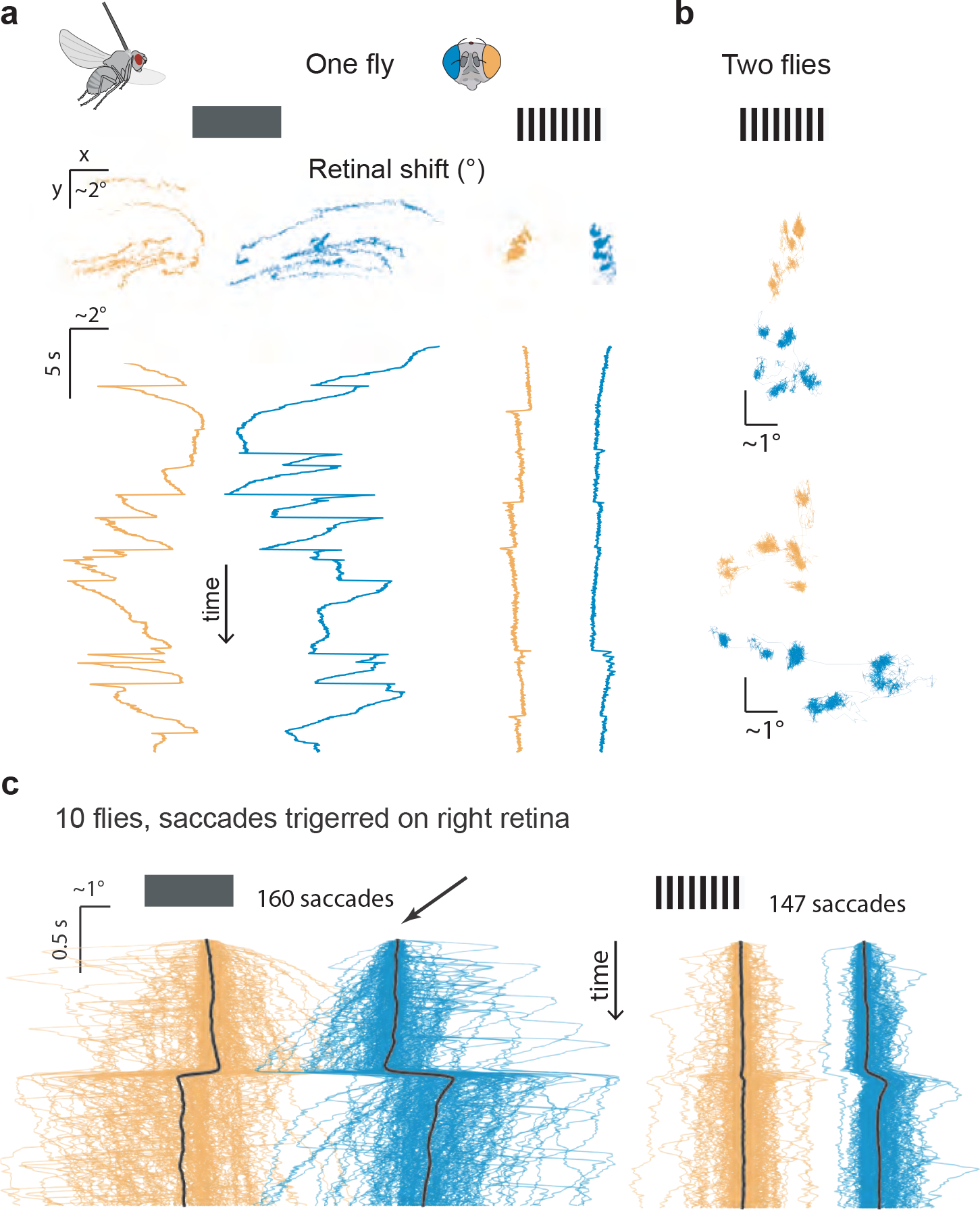
*Drosophila* spontaneously move their retina, often in a saccadic fashion. **(a)** Spontaneous retinal movements in a flying fly viewing a dark screen (left) or a stationary vertical grating (right). Left eye: orange throughout. Right eye: blue throughout. Top: x-y plot of pseudopupil movements. Bottom: horizontal (x) component of the pseudopupil movement over time. **(b)** X-Y plots of both retinas in two more flies viewing a stationary grating (20 s). **(c)** We isolated moments in which the right eye generated large saccades in the x dimension and averaged the x movements of the right and left eye around those times for our population, in the context of either a dark screen or a stationary, vertical grating.

Many animals move their eyes not only in response to external visual motion, but also seemingly spontaneously. We found that this is true in *Drosophila* as well (e.g., Extended Data Movie 8). Tracking the deep pseudopupil of a tethered, flying fly in the context of a stationary panoramic grating, we observed that the fly would keep its retina at a stable position for some time, move it rapidly to a new stable position, hold it there, then rapidly move it again, and so on (Fig. 4a, right). The sample fly in Figure 4a (right) performed fast retinal movements (Methods), or saccades, with amplitudes typically below 1°. A different tethered, flying fly performed larger saccades in the context of the same panoramic stimulus (Fig. 4b). In darkness, the pseudopupil of the sample fly in Figure 4a had an x-y position that drifted extensively, and the fly also exhibited larger saccades of up to ~5° (Fig. 4a, left). These data suggest that flying flies need structured visual input to keep their retinas stable.

We quantified the saccades flying flies performed in the context of a stationary grating and in darkness by detecting large saccades in one eye and plotting these alongside the concomitant retinal movement in the other eye (Fig. 4c). We observed significantly larger saccade amplitudes in flying flies in the context of a dark screen than with vertical gratings (means +/− standard deviation: 2.6° +/− 1.6 ° vs. 0.9° +/− 0.4) (Fig. 4c).

## Small saccades activate visual neurons

Spontaneous retinal saccades often have amplitudes below 1°. Do such small movements of the retina have any impact on visual processing? We performed whole-cell patch-clamp recordings from the horizontal and vertical system cells: motion-sensitive interneurons in the fly’s lobula plate^38,39^. We observed direction-selective depolarizations and hyperpolarizations in response to tiny retinal movements (Extended Data Fig. 8), demonstrating that even the smallest retinal saccades are registered by the visual system (see Supplementary Text). Voltage responses to saccades were often small, however, in some flies we observed visual responses with magnitudes approaching the cell’s full dynamic range as estimated by responses to drifting gratings (Extended Data Fig. 8b).

## Retinal movements in gap crossing

We have shown that retinal movements impact visual-neuron physiology both by shifting receptive fields and by activating motion-sensitive neurons. How might retinal movements impact fly behavior (beyond improving image stability)?

Past work has argued that flies visually assess the length of a gap in deciding whether and how to cross it^40,41^. Specifically, if a gap is deemed crossable, *Drosophila* perform a set of leg-reaching movements to contact the other side, ultimately pulling themselves over; if a gap is uncrossable, flies are less likely to attempt to cross the gap at all^40^. Motion parallax has been suggested as one depth-estimating mechanism that flies might use in assessing depth during this task^40^. We wondered whether *Drosophila* might perform active retinal movements during gap crossing. We were particularly curious if flies perhaps performed active vergence movements as they crossed the gap; because flies have a binocular overlap zone of ~15° in the frontal visual field, such movements, in principle, could help flies to assess depth via either *binocular triangulation* or *binocular-ruler* mechanisms^2,42^ (see Supplementary Text). Retinal movements during gap crossing could serve other roles as well.

**Figure 5.**
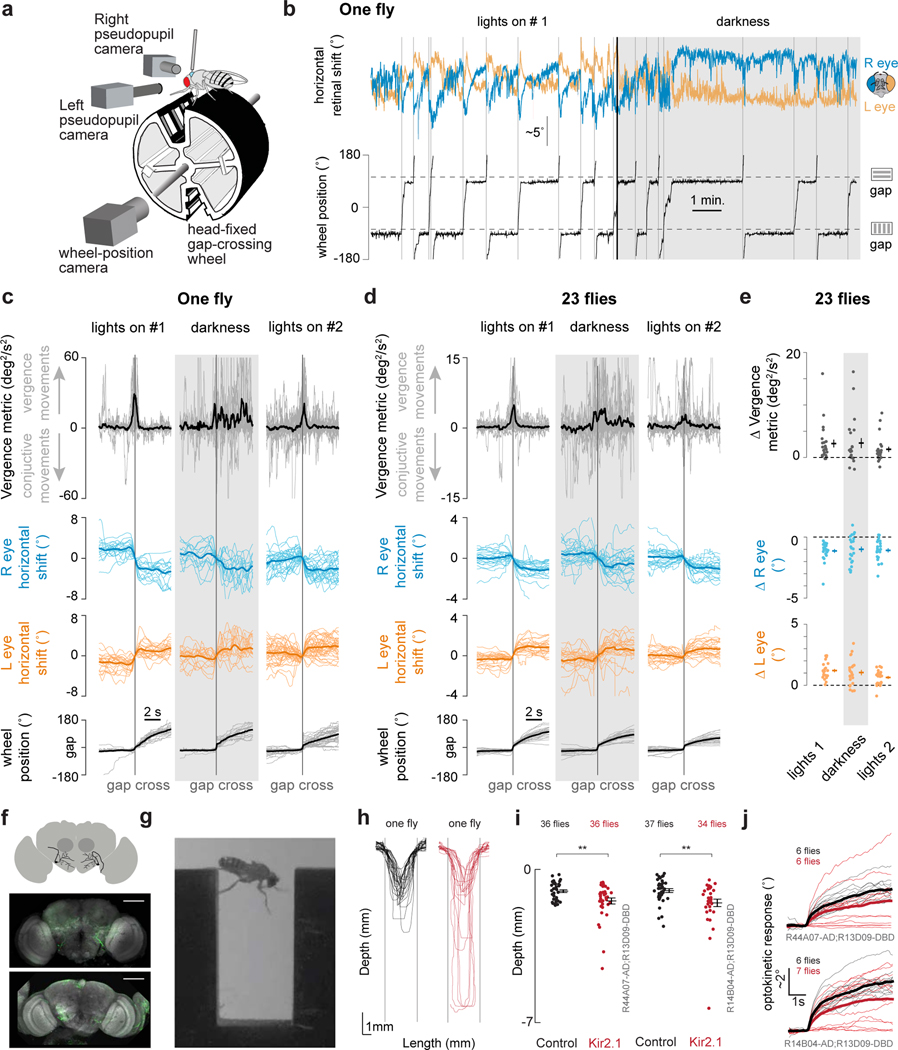
Retinal movements are important for *Drosophila* when crossing gaps. **(a)** Tethered flies walked on a custom, 3D-printed wheel with two, 2.5 mm gaps. One gap had horizontal stripes and the other vertical stripes painted on the walls. Using cameras, we tracked the position of the wheel and the positions of the two retinas. **(b)** Example time series of the right and left deep pseudopupil positions (top) and wheel position (bottom) in a fly that performed particularly large vergence movements at the time of gap crossing. The dotted horizontal lines indicate the threshold position used to detect a gap crossing; vertical grey lines indicate a gap crossing event in the forward direction (Methods). **(c)** Gap-crossing-triggered averages revealed consistent convergent retinal movements at the moment of gap crossing. We plotted the sign-inverted product of the left- and right-eye retinal shifts (Methods) (top) as a metric that goes positive during coincident vergence movements. **(d)** Same as (c) but for a population of 23 flies. **(e)** Quantification of the time traces in (d). For the wheel position and the left- and right-eye retinal shifts, we calculated the mean baseline signal in a 3 s window, starting 5 s before gap crossing and we subtracted this value from the mean signal in a 3 s window starting 2 s after gap crossing. For the vergence metric we subtract the mean signal in a 1 s baseline window starting 2.5 s before gap crossing from the mean signal in a 1 s window surrounding the gap crossing event. All distributions are significantly different from zero (t-test, P<0.05, with a Bonferroni correction for 9 tests), except the vergence-measure distribution in darkness, which has a P value of 0.0083 that is just above the 0.0056 needed after the Bonferroni correction. **(f)** We expressed Kir2.1 in two split GAL4 lines targeting retinal motor neurons. Schematic of the position of the retinal motoneurons, and immunostainings of two split GAL4 lines > Kir2.1-T2A-tdTomato (R44A07-AD;R13D09-DBD top, R14B04-AD;R13D09-DBD bottom). Scale bars: 100 μm). **(g)** Snapshot of a fly crossing a gap, to scale with the plots in h,i. **(h)** Left: x-y trajectories of gap crossings in a control example fly. Right: as on left for an example fly expressing Kir2.1. **(i)** Median of minimum positions for all crosses per fly (dots) together with mean +/− SEM (black lines) for R44A07-AD;R13D09-DBD control (black) and silenced (red) flies, and the equivalent plots for line R414B04-AD;R13D09-DBD. **(j)** Optokinetic responses of both eyes in in control (black) and silenced (red) flies for both split GAL4 lines.

Because we cannot as of yet measure retinal movements in freely moving flies, we developed a head-fixed gap crossing paradigm for *Drosophila*. Pin-tethered flies walked on a wheel that rotated along one axis^43^. The wheel included two, 2.5-mm wide gaps, 180° apart (Figure 5a). One gap had horizontal stripes on the walls and the other had vertical stripes (Methods); data associated with the two gaps have been combined because we noticed no consistent difference in the flies’ behavior across them. We tracked the position of the wheel (Methods) as the flies walked with lights on for 15 min., in complete darkness for 15 min., and with the lights on again for 15 min. On average, flies crossed a gap in the forward direction every 51 s with the lights on and every 67 s in darkness, with considerable variability across individuals; that flies crossed gaps at a lower rate in darkness—alongside control experiments that showed no measurable optokinetic responses to a physically moving grating in darkness—argues that the flies could not see during the lights-off epoch (Extended Data Fig. 9). Whereas freely walking flies are unlikely to cross a gap that they cannot see, pin-tethered flies do so extensively, perhaps because their only navigational alternative, being rigidly tethered, is to walk backwards.

The example fly in Figure 5b consistently exhibited convergent pseudopupil movements (i.e., divergent optical axis movements across the two eyes) when crossing gaps (Fig. 5b, vertical grey lines) (Extended Data Movies 9–11). This observation held when we averaged retinal movements across all gap crossing events for this fly (Fig. 5c,e) and for a population of 23 flies (Fig. 5d,e). We observed similar vergence movements with the lights on and in darkness, albeit with more variability in darkness. That tethered flies made vergence retinal movements when crossing gaps in darkness argues that these movements reflect an active vision strategy rather than sensory responses to visual features of the gap. While moving the retina to improve visual perception is futile in darkness it is still attempted, likely as a reflex, much like a human would likely move their eyes in reflexive ways if forced to perform a visually guided task in darkness.

If retinal movements contribute to gap crossing, then impairing their dynamics might be expected to alter gap-crossing trajectories. Using two different split-Gal4 lines, we muted electrical signaling in retinal motor neurons by expressing in them a modified-mouse Kir2.1 ion channel; genetic-background matched controls expressed a mutated, non-conducting form of this channel^43^. We tracked freely walking flies crossing 3.5-mm long gaps. Gaps of this length are challenging but crossable in free behavior^40^ (Fig. 5g,h). Control flies predominantly crossed the gap near the top, whereas experimental flies were more likely to walk down the near wall before crossing, yielding a statistically lower mean crossing height (y) for both experimental genotypes (y = −1.4 +/−0.1 mm, mean +− SEM, in silenced flies compared to −1.0 + −0.1 mm in control flies in one line, and y = −1.5 +/− 0.2 mm versus y = −1.0+/− 0.1mm in the second line; p<0.01 for both split-GAL4 lines, Welch Test) (Fig. 5i). A quantitative phenotype—i.e., 40–50% lower y-values—is consistent with the fact that expression of Kir2.1 yielded only a partial, ~33–35%, impairment to the magnitude of optokinetic retinal movements in both genotypes (Fig. 5j). Improved split-Gal4 lines (should they be possible to generate) that target the set of motor neurons more comprehensively and with higher transgene expression levels, should allow one to test the effect of more complete silencing of retinal movements on behavior in the future. Regardless, these data demonstrate that normal retinal-motor-system physiology is needed for flies to cross challenging gaps in a canonical fashion.

## Discussion

Retinal optokinetic responses (Fig. 3) and spontaneous saccades (Fig. 4) in *Drosophila* conspicuously resemble human eye movements made in similar contexts. That the dynamics of an insect retina, actuated by two muscles, shows similarities to those of the vertebrate eye, actuated by six muscles of different origin, reveals a remarkable example of homoplasy in animal vision. *Drosophila* compound eyes have no equivalent of an area centralis or fovea, and many primate saccades are thought to be related to the act of foveation. Instead of foveation, spontaneous retinal saccades in flies could help to refresh the visual image in the face of receptor adaptation and we speculate that they may also improve the ability of flies to perceive fine spatial features by dynamically realigning the photoreceptors in relation to those features. Retinal movements, in principle, could also contribute to other functions in *Drosophila* such as spatial attention and visual object recognition, alongside providing a potential proxy measure for whether flies are awake, asleep, or experiencing varying levels of arousal.

We found the flies reliably perform vergence retinal movements when crossing gaps (Fig. 5). One function these movements might serve is to estimate the length of the gap via a binocular-ruler mechanism^42^ (see Supplementary Text). Notably, LC14 neurons (Fig. 2) appear particularly well suited for implementing such a function^11,19,20,44^ because they (1) have front-facing receptive fields in the binocular overlap region, (2) they are particularly sensitive to visual features like edges or small objects and (3) they have axons that interconnect the two visual lobes, providing an anatomical substrate for a binocular comparison. The advanced experimental toolkit in *Drosophila* should allow us to test this idea, and many related ideas on the role of retinal movements, in the coming years. More generally, the discovery of retinal movements in *Drosophila*—a premier genetic model organism—should allow experimenters to develop a deep understanding of how active vision works in one species^10–12,45–50^, serving as an example for many others down the road.

## Methods

Methods are provided in the Supplementary Information.

## Extended Data

**Extended Data Figure 1:**
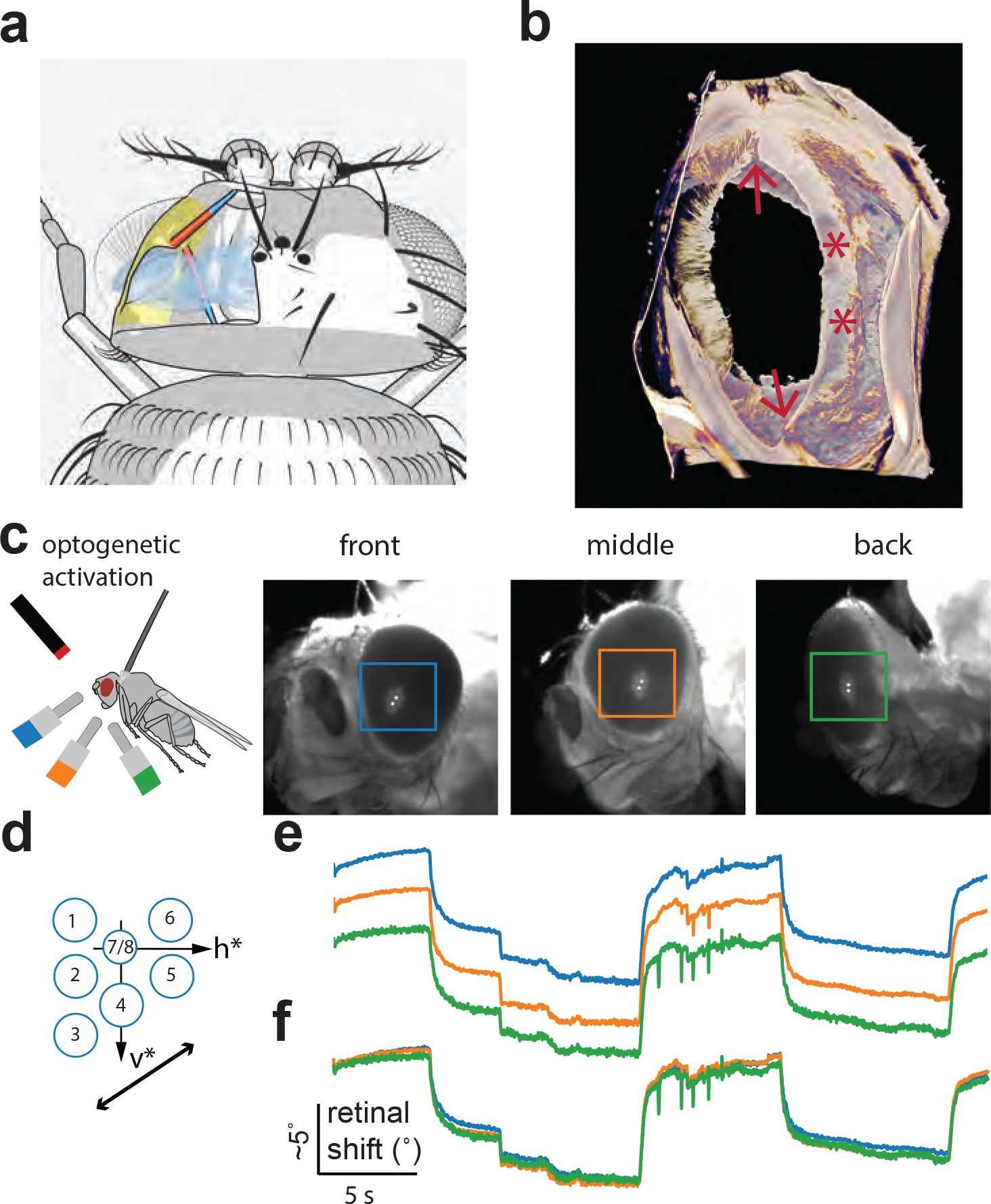
Retinal muscles that attach to the front of the orbital ridge can move the entire retinal sheet coherently. **(a)** Schematic of the attachment of the retinal eye muscles to the orbital ridge in the *Drosophila* head (for an animated version see Extended Data Movie 1). **(b)** Staining of a part of the *Drosophila* head including the eye and the orbital ridge in a cleared specimen using Calcofluor White. Soft tissue was removed proteolytically. It reveals the vesica piscis-shaped opening of the orbital ridge and shows strongly sclerotized parts in the frontal region, where the two muscles attach (red stars), as well as two discontinuities on the dorsal and ventral poles (arrows). One possibility is that these discontinuities decouple the front of the orbital ridge from the back, mechanically. This may allow muscles that pull the front of the retina to also move the rear by the same amount through internal cohesion within a stiff set of ommatidia, rather than through a force vector that dissipates over space from front to back. Alternatively, the inhomogeneities at the top and bottom of the orbital ridge could act as a fulcra or pivot points, leading to the rear part of the orbital ridge to move outward when the frontal part moves inward (towards the midline), which could aid coherent motion of the retina. We will test these models in biomechanical studies in the future. For an animated version of the Calcofluor White image stack see Extended Data Movie 2. **(c)** We measured retinal movements using three cameras pointing at three different positions in one eye. To induce large movements, we optogenetically activated the retinal motoneurons. We expressed CsChrimson in a split-GAL4 line (w+;R44A07-AD;R13D09-DBD) and focused red light onto a spot between the fly eyes (Methods). The three plots to the right show the views of the three different cameras: we overlaid images before and after optogenetic activation and plotted the tracked centroid on top of the pseudopupil (white) to illustrate the retinal movements. **(d)** Optogenetic activation in this fly yielded movements roughly along the axis connecting photoreceptors 3–5, and we used this motion direction (dark arrow) for comparison across the different positions on the facet eye in (e). Schematic modified from Stavenga (1979)^51^ **(e)** Traces of the pseudopupil measured in front (blue), in the middle (orange) and in the back (green) of the fly eye. **(f)** Same traces as above but shifted in y (by hand) to illustrate the coherent motion of the pseudopupil across the eye.

**Extended Data Figure 2:**
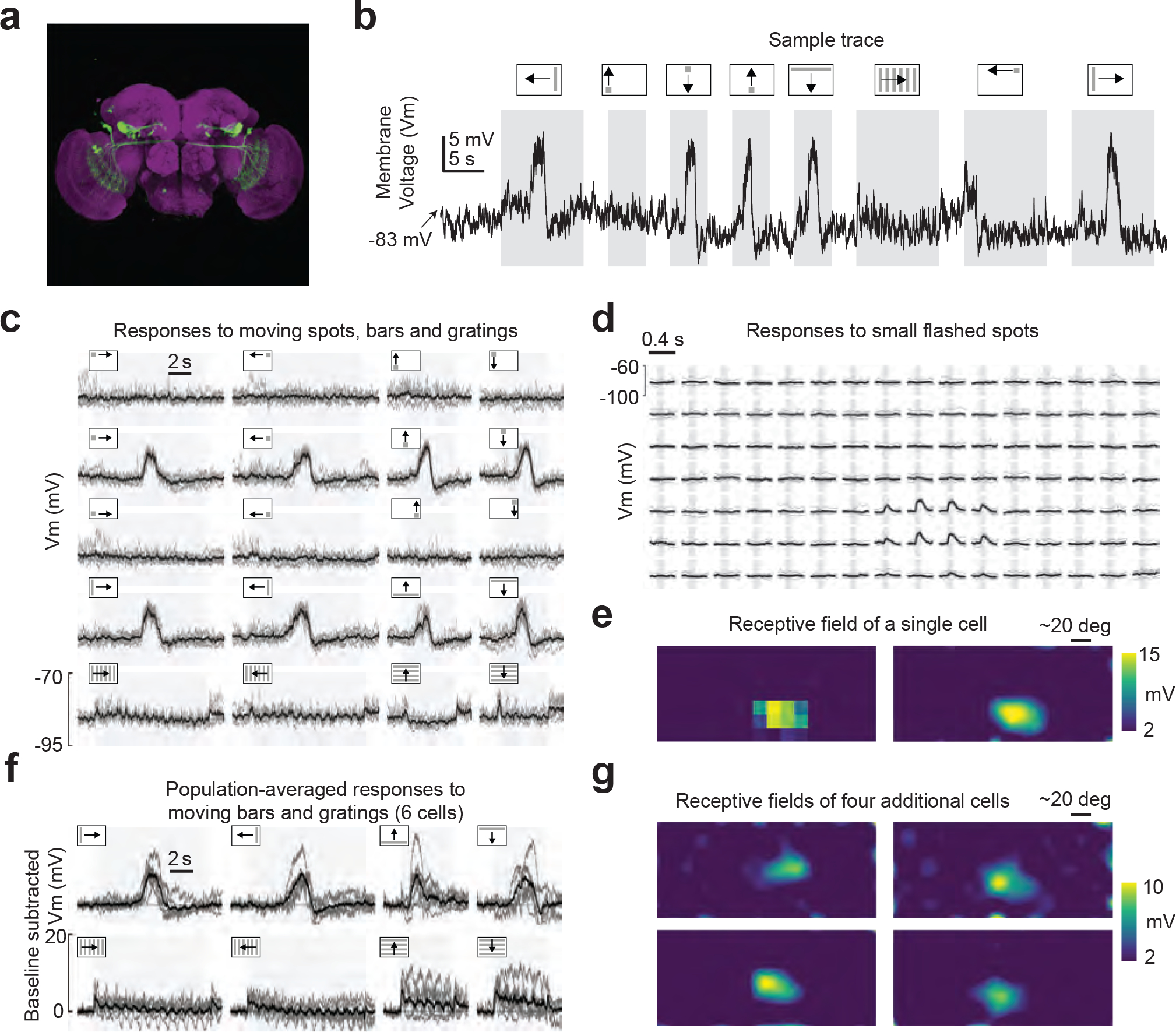
Electrophysiological recordings from mDCNs reveal that they have small, front facing receptive fields and that they respond strongly, in a non-direction-selective manner, to moving spots and bars. **(a)** GFP expression in the VT37804-GAL4 Line shows LC14 cells, which connect the lobula on one side to the contralateral lobula and medulla. Additional off-target expression in the anterior optic tubercle is also visible. **(b)** Example trace showing the mDCN membrane voltage (*Vm*) while it is modulated by a variety of visual stimuli (bars, spots and gratings). **(c)** Membrane potential responses from a single cell reveal strong, non-directional responses to moving spots and bars in a restricted portion of the visual field. Light gray: single trials. Black: mean response. **(d)** Single cell response to 100 ms spot flashes (gray region) on the LED screen. Light gray: single flashes. Black: mean response. **(e)** Heat map image representation of an LC14 cell’s receptive field (same cell as shown in d). **(f)** Population-averaged responses to moving bars and gratings (six cells) reveal more consistent responses to bars than gratings. **(g)** Heat-map representations of four more LC14 cells’ receptive fields. (Bottom left plot shows same data as Fig. 2b)

**Extended Data Figure 3:**
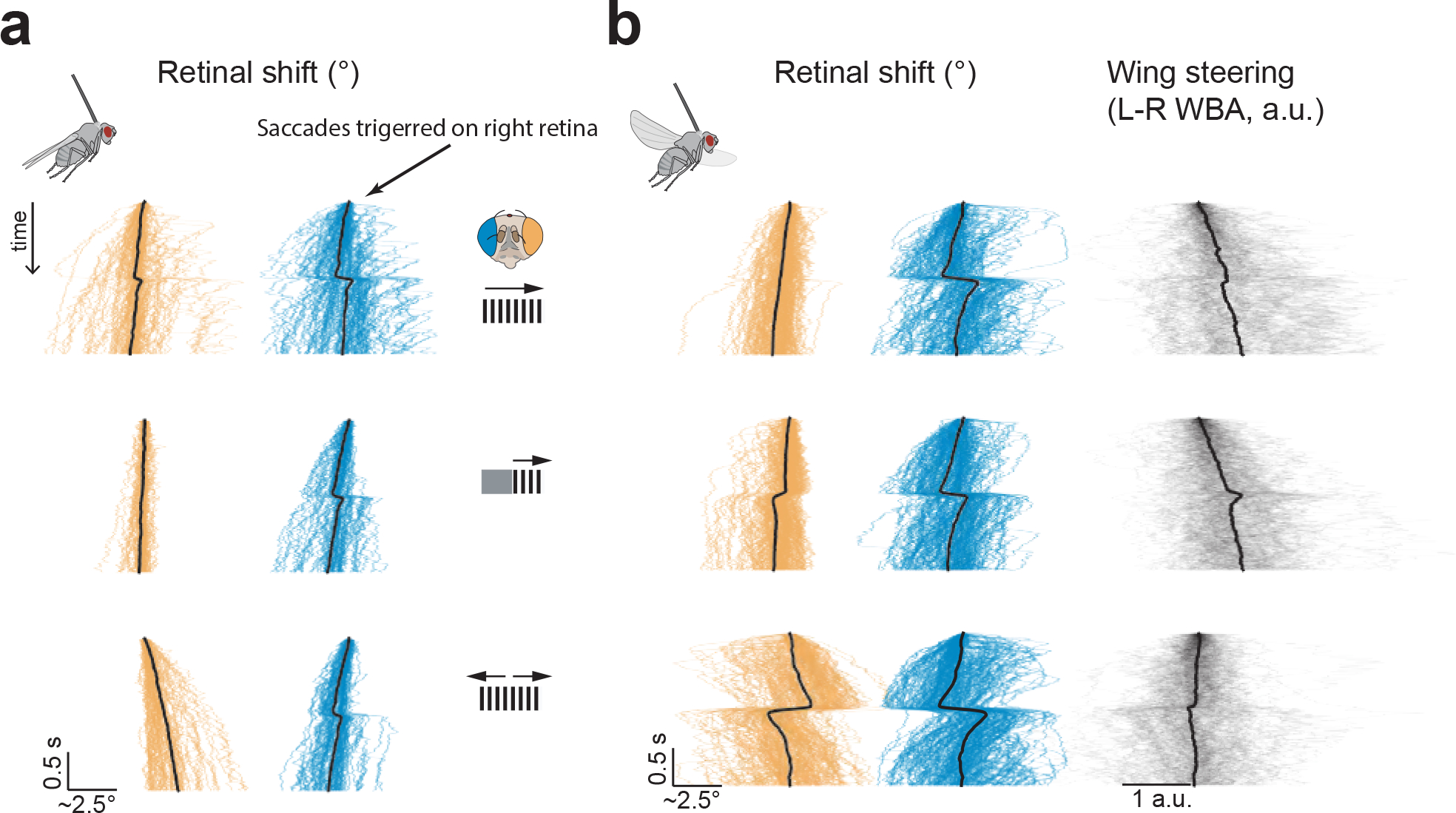
Optokinetic retinal tracking is interspersed with (nystagmus-like) counter-saccades and the largest counter-saccade magnitudes are observed in flight. **(a)** We isolated large saccades of the right retina with a simple, threshold-crossing algorithm and plotted these alongside the concomitant retinal movements of the left retina in 10 quiescent (i.e., non-flying) flies. Data are shown in the context of full-field rightward grating motion (top) (87 saccades), rightward motion in the right visual hemisphere (middle) (56 saccades), and bilateral front-back-motion (bottom) (66 saccades). **(b)** Same as panel a but during tethered flight. We also show the left-minus-right wing beat amplitude (L–R WBA) of the flies, with rightward deflections indicating a rightward steering response and vice versa (top, middle and bottom traces include 122, 136 and 152 saccades respectively).

**Extended Data Figure 4:**
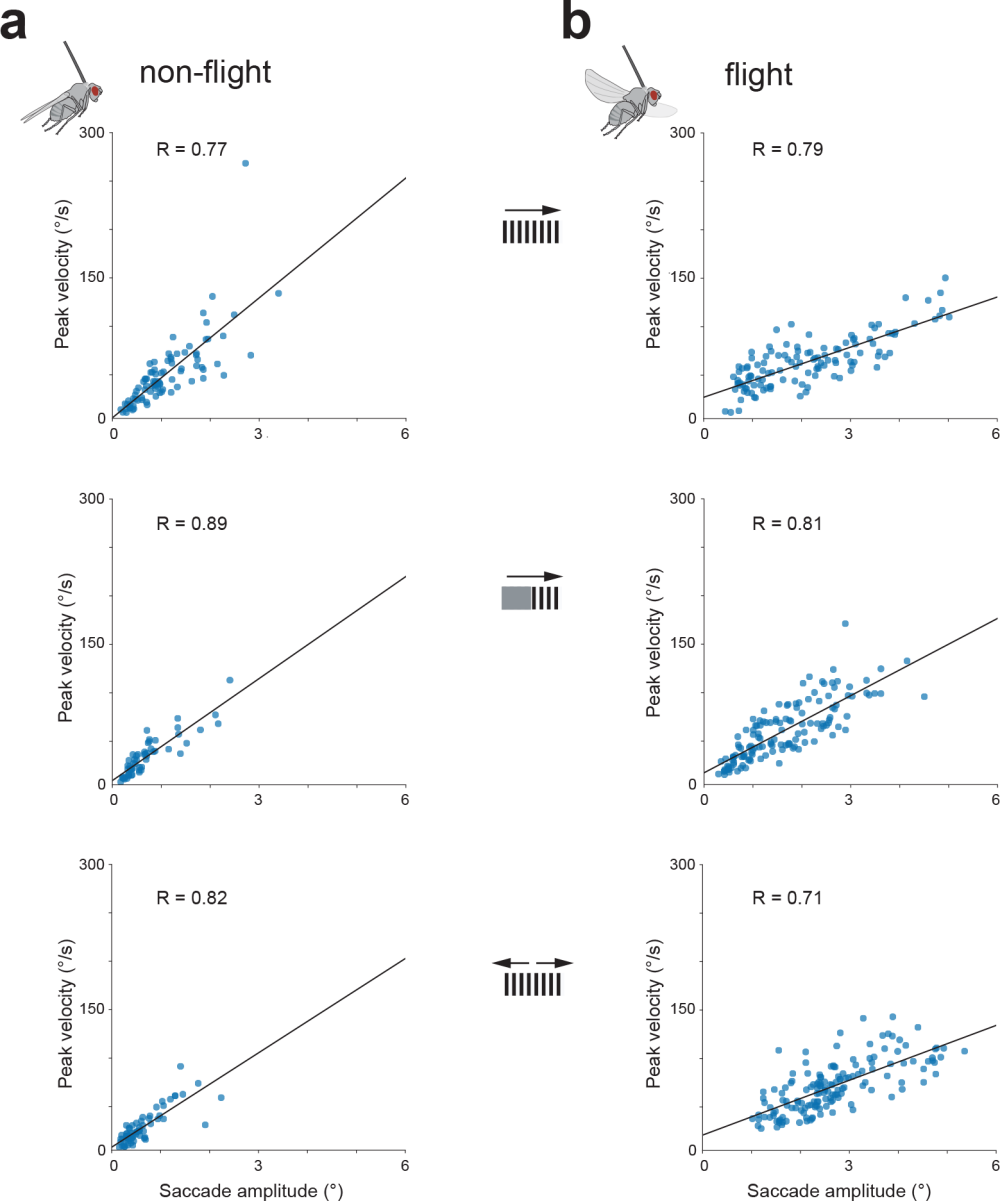
Retinal saccade magnitudes and peak velocities are tightly correlated, akin to the *main sequence* in human saccades. **(a)** Saccade peak velocity plotted against saccade amplitude of the saccades of the right retina from Extended Data Fig. 3a. Data are shown in the context of full-field rightward grating motion (top) (87 saccades), rightward motion in the right visual hemisphere (middle) (56 saccades), and bilateral front-back-motion (bottom) (66 saccades). **(b)** Same as panel a but for the saccades of the right retina from Extended Data Fig. 3b, which occurred during tethered flight (top, middle and bottom traces include 122, 136 and 152 saccades respectively). The mean saccade amplitudes for flight and quiescence were 2.6° and 1.1° for full-field rightward motion, 1.6° and 0.7° for rightward motion in the right hemisphere and 2.7° vs 0.6° deg for bilateral front-to-back motion. All saccade-magnitude differences between flight and quiescence were highly significant (p<10^−10^, two-sided t-test).

**Extended Data Figure 5:**
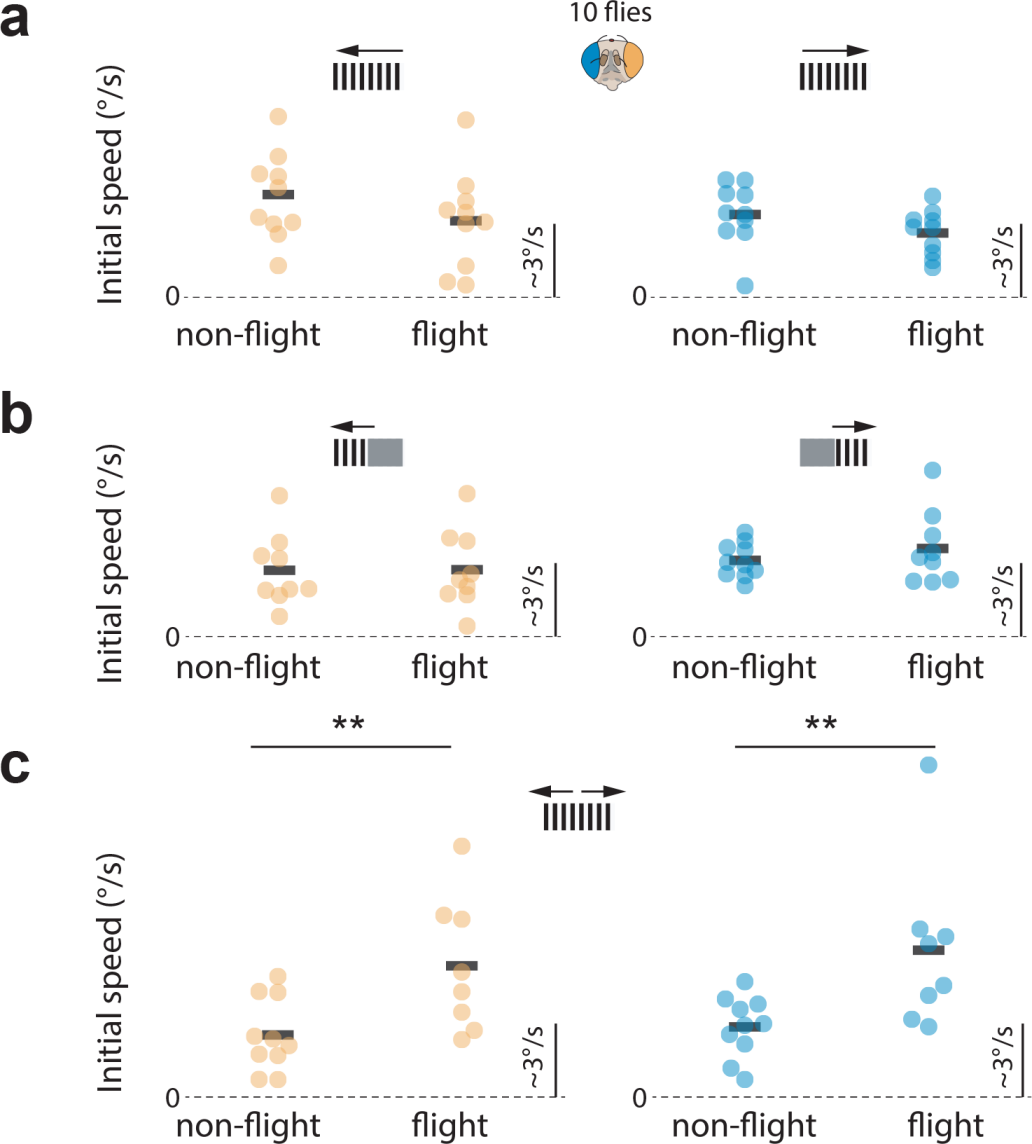
The gain of optokinetic tracking is below unity and was significantly modulated by flight for bilateral front-to-back motion. **(a)** The initial pseudopupil velocity for full-field 15°/s leftward grating motion when analyzing the left eye (orange) and for full-field 15°/s rightward grating motion when analyzing the right eye (blue). Data for optokinetic responses made during quiescence and tethered flight are shown separately. Each point represents data from one fly. **(b)** Initial pseudopupil velocities for unilateral motion stimuli: 15°/s grating motion in the left side for the left eye and right side for the right eye. **(c)** Initial pseudopupil velocities for bilateral front-back-motion. The initial pseudopupil velocities during flight and quiescence were not significantly different for rotational stimuli **(a, b)** but they were significantly bigger in flight for bilateral front-to-back motion, which simulates forward translation (two-sided Wilcoxon signed-rank test, left eye: p=0.005, right eye: p=0.007).

**Extended Data Figure 6:**
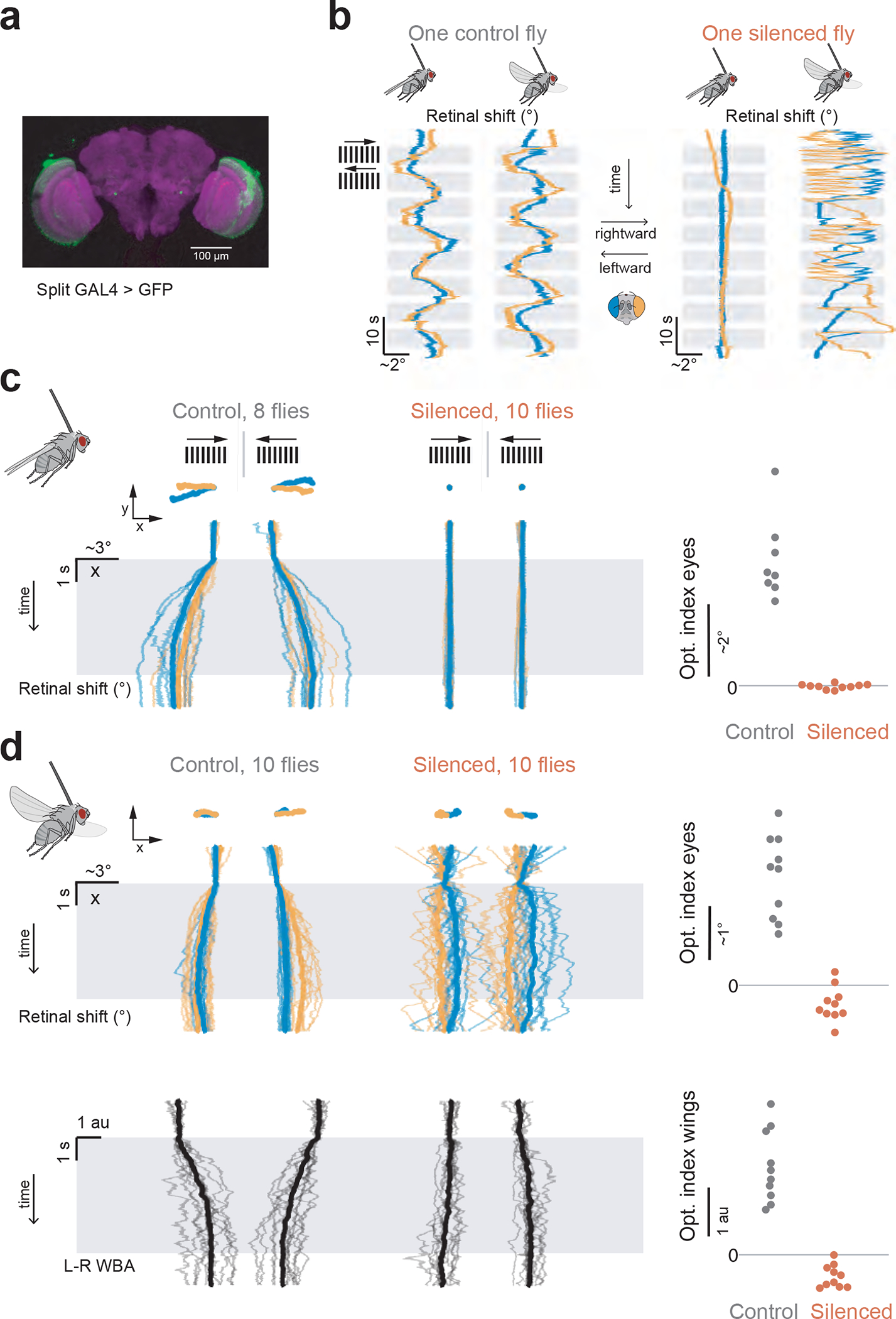
Silencing neurons in the early fly visual system abolishes optokinetic responses to visual motion but preserves spontaneous retinal movements in flying flies. **(a)** Immuno-stain of a split-GAL4 line labelling L1-L4 visual neurons (green) and neuropil (anti-*brp*, magenta) **(b)** Example traces showing retinal movement in a control fly, expressing inactive tetanus toxin in the L1-L4 cells, and an experimental fly, expressing active tetanus toxin in the same neurons, in flight and non-flight. All behavioral responses were made in the context of a full-field grating rotating at 15°/s (1 Hz temporal frequency). **(c)** Single fly averaged (thin lines) and population averaged (thick line) retinal responses for 8 control flies and 10 experimental flies during non-flight/quiescence. Right panel: Optomotor index (Supplementary Information) quantifying the response strength to visual motion in the expected optokinetic direction. **(d)** Same as panel c, but in flying flies. Black: Left-minus-right wingbeat amplitude (L–R WBA).

**Extended Data Figure 7:**
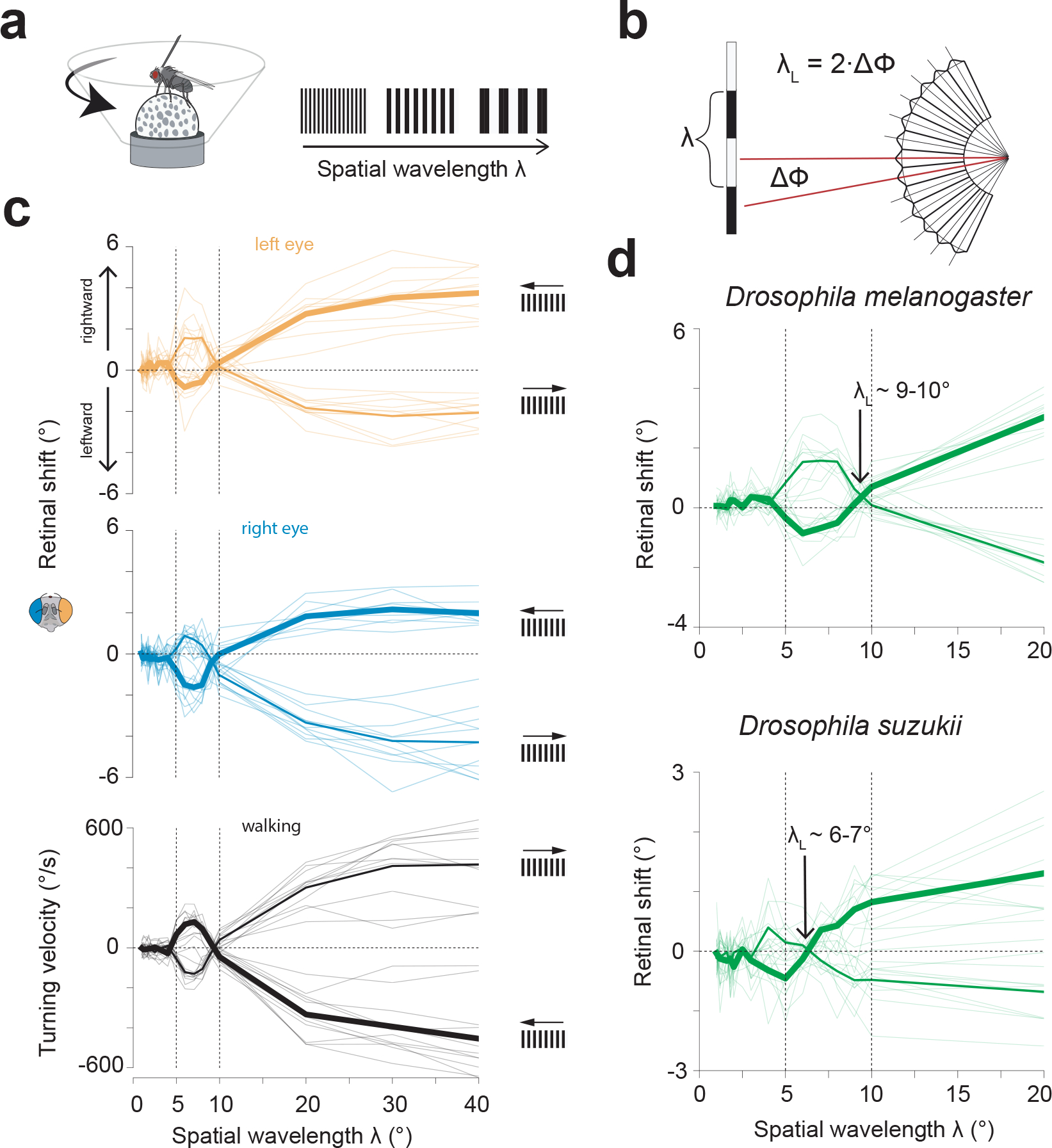
In both *D. melanogaster* and *D. suzukii*, the sign and magnitude of retinal optokinetic responses and walking optomotor responses are consistent with non-hyperacute sampling of the visual world by the fly retina. **(a)** Experimental setup: pin-tethered flies walked on floating ball. We recorded the ball’s rotations as a readout of the flies’ turning velocity (black) alongside the displacements of the deep pseudopupil (left: orange, right: blue). Visual stimuli were presented on a conical screen using a projector and consisted of full-field rotating gratings at varying spatial wavelengths (always 4 Hz temporal frequency) for 5 s. **(b)** The inter-ommatidial angle Δϕ of the compound eye limits the spatial wavelength λ of a grating that can be properly resolved (modified from Land 1997^52^). Below the cut-off wavelength of λ_L_ = 2·Δϕ, direction-selective motion responses of the visual system are predicted to invert due to spatial aliasing^52,53^. **(c)** Top and middle: displacements of the left and right pseudopupils during the stimulus period as a function of the grating wavelength, λ (1 to 40°) for left- and rightward motion. Bottom: concomitant average walking velocity during the stimulus period. Thin lines are the trial-averages from 11 single flies. Thick lines are population averages. Note the sign inversion in the range of λ=5–10° for both the retinal optokinetic reflex and the walking optomotor response. **(d)** Average responses for both retinas for front-to-back (thicker line) and back-to-front motion for *Drosophila melanogaster* (top, data as in b) in comparison to *Drosophila suzukii* (bottom). *D. suzukii* showed inverted responses at smaller wavelengths than *D. melanogaster*, i.e. had a higher spatial acuity, consistent with the fact that *D. suzukii* have approximately twice the number of ommatidia as *D. melanogaster*^54^. The sign-inversion of behavioral responses around the critical wavelength inferred from the optics of the eye argues that any retinal movements flies are making in the context of moving gratings are not allowing them to perceive the motion direction of fine gratings better than would be expected from the first-order optics of the eye. The lack of evidence for hyperacuity in this context does not exclude it existing in other circumstances.

**Extended Data Figure 8.**
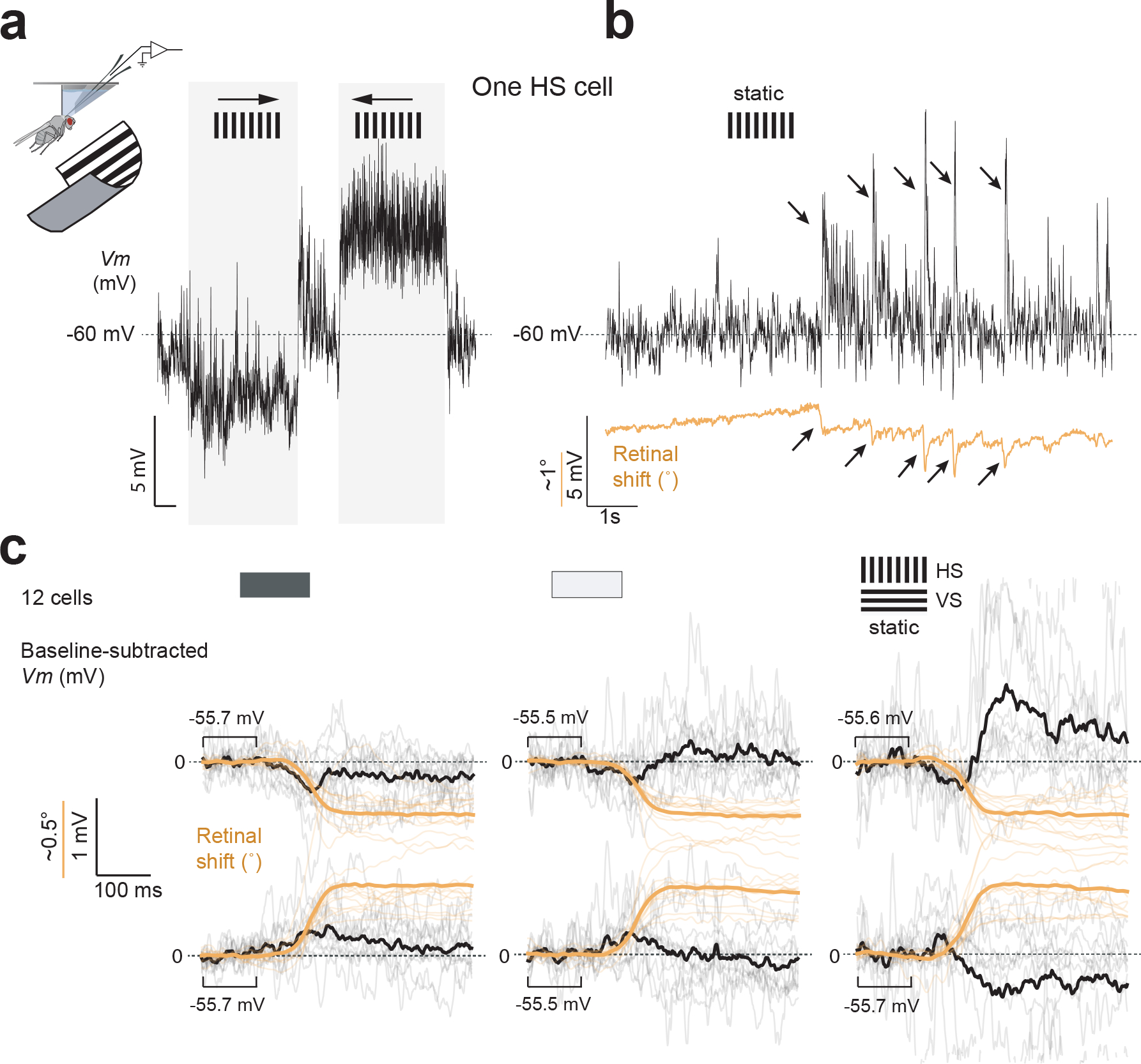
Visual neurons activate during spontaneous saccades with direction-selective responses. **(a)** Left: sample *Vm* responses of an HS cell on the left side of the brain to rightward followed by leftward grating motion (1 Hz temporal frequency). **(b)** *Vm* of the same cell, alongside the x-movements of the left retina (orange), in the context a stationary vertical grating. Arrows indicate moments of spontaneous saccades. **(c)** Trial-averaged *Vm* of single flies (gray, N=12 HS or VS cells) and population averaged *Vm* (black) for left-eye retinal saccades (orange). Data from a dark arena (left), a uniformly lit arena (middle), and a stationary grating (right) are shown. Top plots shown downward retinal movements for VS cells and leftward retinal movements for HS cells, which should produce visual motion in the preferred direction. Bottom plots show the opposite, null-direction retinal movements. The direction-selective responses to gratings argue that HS and VS cells respond to the visual motion induced on the retina by < 1° eye movements. The weak response to eye movements in darkness, or with a uniformly lit screen, is opposite in sign to that observed with a grating, which may represent an efference copy of the predicted motion signal arriving to HS/VS cells with each eye movement. This efference copy is potentially superseded by the actual, grating motion input with a high contrast grating, in the rightmost column.

**Extended Data Figure 9.**
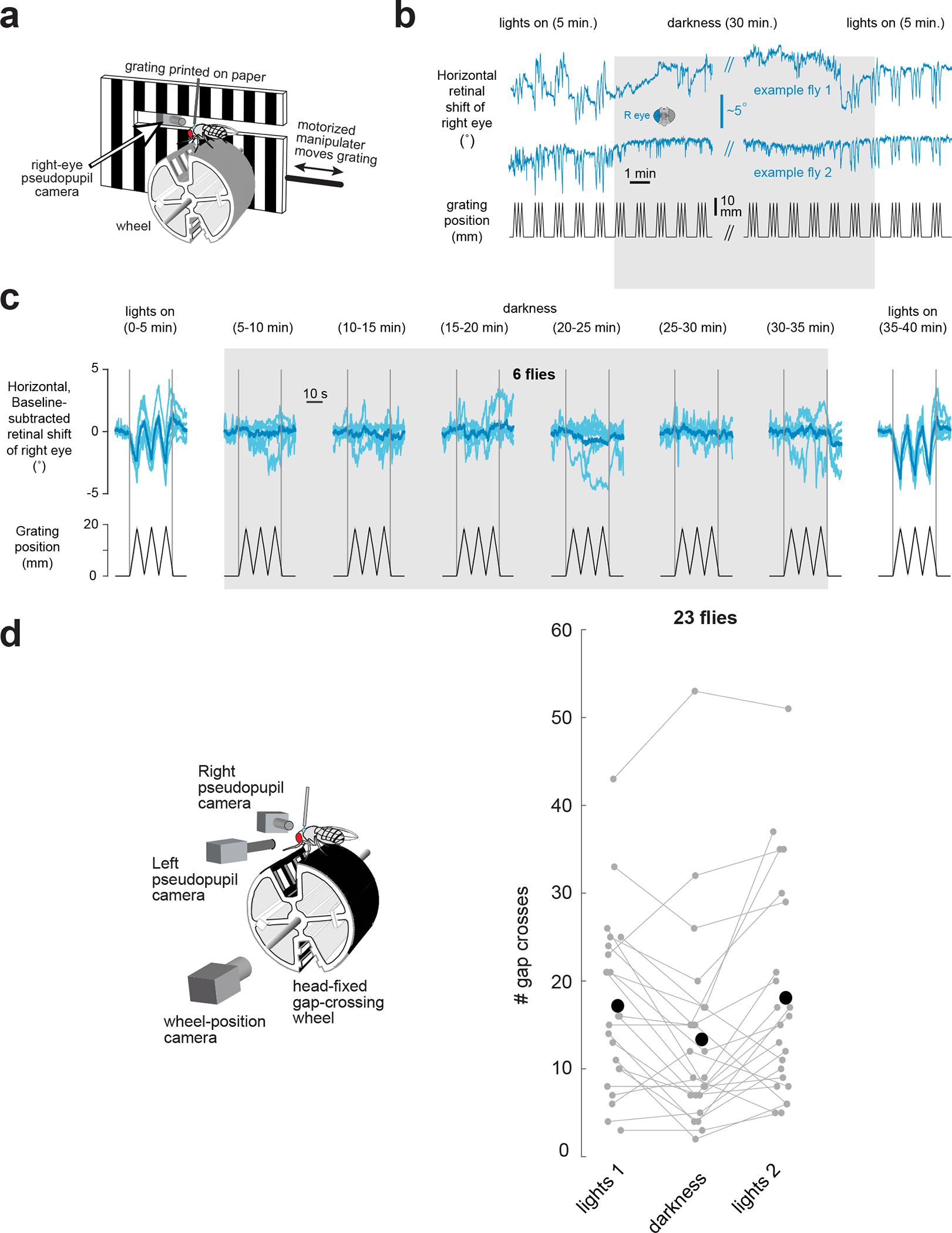
Evidence that flies are genuinely in the dark during the lights-off epoch of the gap crossing experiments. **(a)** Flies walking on the gap-crossing wheel (Fig. 5) were presented with a grating printed on paper that was physically moved back and forth in front of the right eye with a motorized manipulator. A small slit in the printed grating allowed us to slide an InfiniStix lens through it, abutting the fly’s right eye, to track the deep pseudopupil. **(b)** Example traces showing the horizontal shift of the right retina in two flies (blue) together with the grating position (black). **(c)** We observed a clear optokinetic response with the lights on, but not during darkness, demonstrating that there was genuinely no light available for flies to see with the lights off, even after being dark adapted for 30 minutes. Light blue: single flies. Dark blue: population mean. Five repetitions of the grating’s movement were presented and averaged for each fly, in each 5-min. epoch shown. **(d)** We quantified the number of gap crossings from the data presented in Fig. 5 and observed a ~30%, statistically significant, drop in the rate of gap crossing during the dark period (t-test, p = 0.013 when comparing lights on #1 with darkness and p = 0.0056 when comparing lights on #2 with darkness).

**Extended Data Figure 10.**
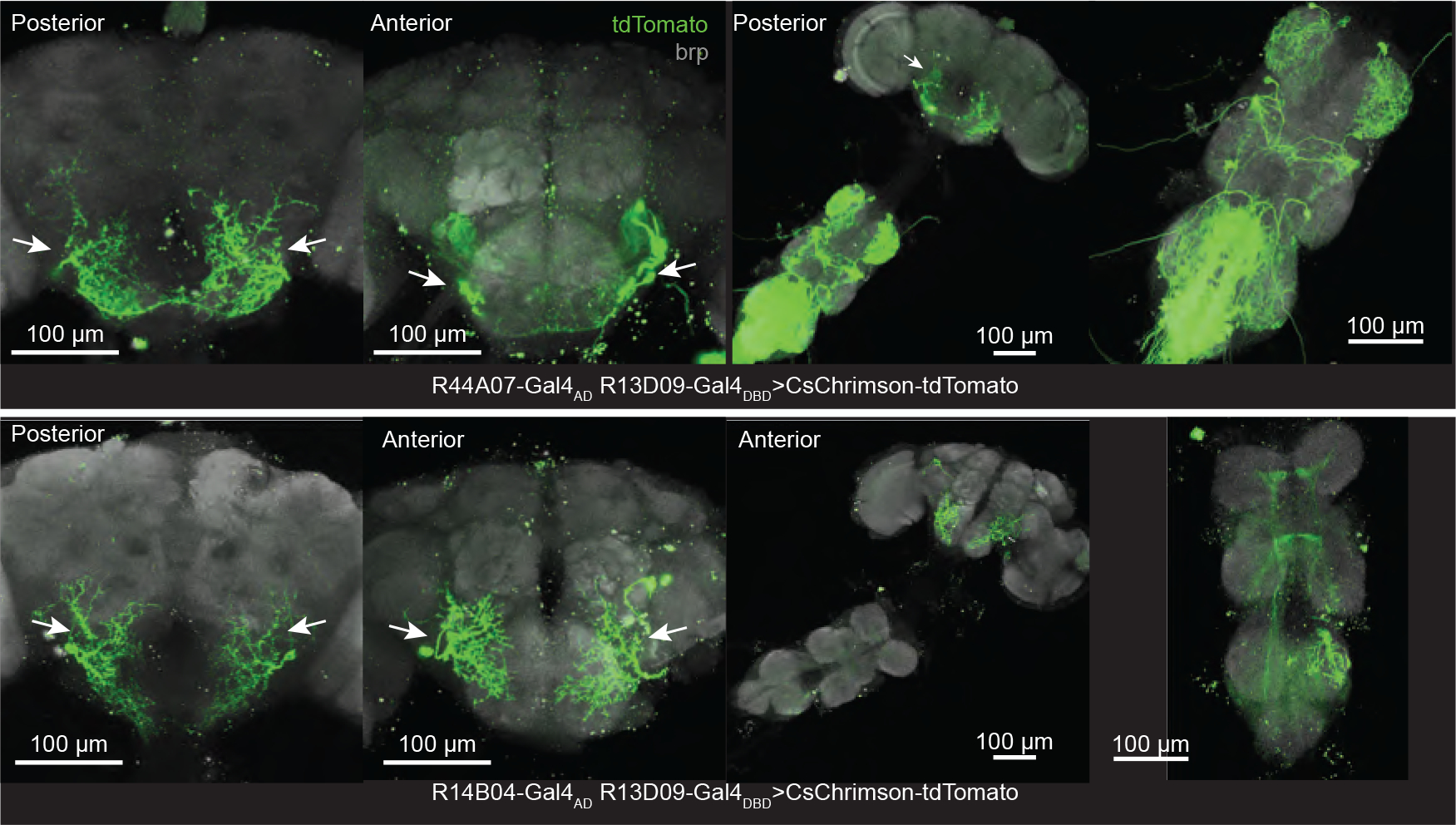
Anatomical characterization of two the split GAL4 lines used for silencing retinal motor neurons. We visualized expression in these two split-Gal4 lines by driving CsChrimson-tdTomato in R44A07-AD;R13D09-DBD (top) and R414B04-AD;R13D09-DBD (bottom). Maximum z-projections of the brain are shown over roughly the posterior and anterior halves to better visualize the branching. VNC maximum projections are shown over the full stack. The green cells on the right and left side of the SEZ (arrows) are retinal motor neurons, based on their dendritic arborization, location of cell body and their axons leaving the brain just below the antennal lobe. We could optogenetically induce retinal movements via expression of CsChrimson expression in both split lines.

## Supplementary Material

Video 1 **Extended Data Movie 1:** Schematic of the attachment of the retinal eye muscles to the orbital ridge in the *Drosophila* head.

Video 11 **Extended Data Movies 11:** Examples of retinal movements during tethered gap crossings (0.25x speed).

Video 10 **Extended Data Movies 10:** Examples of retinal movements during tethered gap crossings (0.25x speed).

Video 9 **Extended Data Movies 9:** Examples of retinal movements during tethered gap crossings (0.25x speed).

Video 8 **Extended Data Movie 8:** Spontaneous retinal movements in a pin-tethered, flying fly in the context of varying, stationary visual scenes (2x speed).

Video 7 **Extended Data Movie 7:** Retinal movements in response to unilateral visual motion in a pin-tethered, non-flying (i.e. quiescent) fly (4x speed).

Video 4 **Extended Data Movie 4:** Simultaneous tracking of photoreceptor tips via a water-immersion objective and the deep pseudopupil via an air lens.

Video 3 **Extended Data Movie 3:** 3D rendering of a portion of the fly head, near one eye, showing immunohistochemical labeling of muscles (red) and chitin (blue).

Video 6 **Extended Data Movie 6:** Retinal movements and wing movements in response to panoramic visual motion in a pin-tethered, flying fly (4x speed).

Video 5 **Extended Data Movie 5:** Retinal movements in response to panoramic visual motion in a pin-tethered, non-flying (i.e. quiescent) fly (4x speed).

Video 2 **Extended Data Movie 2:** Animated stack highlighting the orbital ridge.

1

## Data Availability

The data shown in the main figures are available at https://doi.org/10.6084/m9.figshare.c.6145572. All other data generated in this study are available from the corresponding authors upon request. Custom-written software used to track the fly retina in real time is available at https://github.com/MaimonLab/EyeTrackerForm. Additional code is available upon request from the corresponding authors.
