# Supplementary material for: Muscles that move the retina augment compound-eye vision in Drosophila
Source: Nature. Author manuscript; Available in PMC 2023 Apr 14. (PMC10103069; doi:10.1038/s41586-022-05317-5)
Supplement: 1 [file NIHMS1879933-supplement-1.pdf]

SUPPLEMENTARY TEXT

We could not detect hyperacuity in a classic optomotor assay.

It has been suggested that photocontraction increases visual acuity beyond the optical limit of the compound eye[1]. These experiments were conducted by measuring flight optomotor responses to rotating squarewave gratings with varying spatial wavelengths. We measured retinal optokinetic responses, alongside walking (Extended Data Fig. 7) or flight (data not shown) optomotor responses to rotating gratings and could not detect hyperacuity—i.e., an ability to detect finer spatial wavelengths than afforded by the standard optical sampling of the compound eye—in these experiments. In both *Drosophila melanogaster* and *Drosophila suzukii* (a species with double the number of ommatidia as *D. melanogaster*[2]) we found a sign inversion of both retinal optokinetic responses and walking optomotor responses around the wavelengths where the regular optical spacing of the compound eye units are expected to yield inverted (aliased) motion signals[3] (Extended Data Fig. 7). At wavelengths finer than this inverted limit, we saw no consistent optomotor responses. Our conclusions are in line with Salem et al.[4] who found that flies cannot stabilize gaze on gratings with spatial wavelengths below the Nyquist limit due to aliasing. It is possible that retinal movements (or other mechanisms) increase fly visual acuity in other tasks, but we found no supportive evidence in the context of rotating gratings.

Photocontraction vs. muscular retinal movements.

Recent work has argued for retinal movements in *Drosophila* via a non-muscular mechanism, where phototransduction yields physical movements of the receptors absorbing the photons (i.e., *photocontraction*). Spontaneous retinal movements (e.g., the data in Figure 4 and Figure 5) occur in the absence of acute visual stimulation—particularly the movements we observe in complete darkness—and these obviously cannot be explained by photocontraction (which depend on the absorption of photons by photoreceptors). The optokinetic reflex (Fig. 3) on the other hand is elicited by visual stimulation. Could this behavior be due to photocontraction? We think not, for the following reasons. First, we found that retinal movements elicited by moving gratings (Fig. 3) are abolished upon silencing of L1-L4 visual neurons (Extended Data Fig. 6); downstream motion processing should be impaired with this perturbation, but photoreceptors and photocontractions should have remained intact. Second, the fact that optokinetic responses are bidirectional means that they require a neural circuit to detect the direction of visual motion, which is not possible for photoreceptors to do alone; in other words, photocontraction should have caused the retina to move in the same direction for leftward and rightward moving gratings, but the retina moved in opposite directions for these two stimuli. Finally, we show that retinal movements invert their sign below the Nyquist sampling limit of the lens array, in coordination with flight turning responses (Extended Data Fig. 7), as expected theoretically[3] for visually guided behavior in *Drosophila*; a retinal-movement mechanism that only considers photoreceptors and phototransduction should not invert sign with aliased motion detection, but should only change magnitude as the photoreceptors of interest catch more or less photons.

Motion-sensitive neurons activate during small retinal movements.

Drosophila viewing a stationary image often performed saccades with amplitudes that were <1°. Do such small movements of the retina have any impact on visual processing? We performed whole-cell patch-clamp recordings from the horizontal and vertical system (HS and VS) cells — motion-sensitive interneurons in the fly's lobula plate. To first order, HS cells depolarize to ipsilateral front-to-back motion and hyperpolarize to ipsilateral back-to-front motion (Extended Data Fig. 8a); VS cells depolarize to downward motion and hyperpolarize to upward motion[5,6]. We recorded the membrane voltage (*Vm*) of HS

and VS cells while simultaneously tracking retinal movements in flies viewing a uniformly dark arena, a uniformly lit arena, or stationary squarewave gratings. All recordings were performed in the left optic lobe and we tracked the left retina (Methods). Retinal movements were generally smaller during electrophysiological recordings— likely because some of the head's cuticle is removed to perform patch-clamp experiments—but the movements retained were sufficient to demonstrate their electrophysiological relevance, as shown next.

In Extended Data Fig. 8a, we show a sample *Vm* response of an HS cell to a vertical grating moving in the preferred and null directions. The same cell in the context of stationary vertical grating (Extended Data Fig. 8b) showed obvious *Vm* fluctuations coinciding with small retinal saccades (arrows). Downward deflections in the eye position trace indicate leftward saccades, that (perhaps counterintuitively) yield leftward, or front-to-back, visual motion on the left retina. These saccades were associated with transient depolarization of the HS cell's *Vm*, the expected sign of response if the neuron were responding to the visual motion generated by these retinal movements.

We detected retinal movements in 12 flies and quantified the concomitant *Vm* changes in HS and VS cells. For HS cell recordings, we considered front-to-back visual motion—resulting from a horizontal leftward retinal movement in the context of a stationary vertical grating—to be the preferred motion direction of the cell and for VS cells we considered downward visual motion—resulting from a downward retinal movement in the context of a stationary horizontal grating—to be the preferred direction of the cell. Movements in the opposite direction were considered null-direction movements. Transforming responses to a null/preferred axis like this allowed us to combine HS and VS recordings into a common plot.

We observed bi-directional saccade-triggered *Vm* changes that were smallest in the context of a dark screen (Extended Data Fig. 8c, left) and largest in the context of a stationary grating (Extended Data Fig. 8c, right). The sign of the relatively large *Vm* change ~100 ms after retinal saccades ($p=0.01$, for preferred direction movements, and $p=0.03$ for null direction movements, two-sided Wilcoxon signed-rank test, Supplementary Information) made in the context of stationary gratings was consistent with direction-selective visual responses to the visual motion generated by retinal movements. In darkness, we noticed very small *Vm* changes ($p=0.01$ and $p=0.005$ for the two saccade directions) that on average, had the opposite sign to the visual responses we observed for an equivalent retinal movement made in the context of visual gratings (Extended Data Fig. 8c). These small *Vm* fluctuations to retinal movements in darkness had the correct sign to reflect either the motor command to move the retina or an *efference copy* of that command: a motor-related input to HS/VS cells (or upstream neurons) that acts to minimize the expected visual response arising from a retinal movement. Indeed, even with a uniformly lit screen (Extended Data Fig. 8f, middle) and the visual grating (Extended Data Fig. 8c, right), one can observe a small initial swing in the *Vm* in the direction of the membrane fluctuation observed in darkness. Thus, these *Vm* traces likely reflect the combination of a motor-related component and a visual response, with at least the visual response's magnitude contingent on the nature of the visual scene. In some cases, we observed very large visual responses to retinal movements made in the context of stationary gratings, with magnitudes that approached each cell's entire dynamic range, as estimated by its responses to drifting gratings (Extended Data Fig. 8 a and b).

A potential *binocular-ruler* mechanism for depth perception
Vergence movements might potentially enable binocular distance estimation via two mechanisms. Classically, it is assumed that the retinal resolution in *Drosophila* is too poor compared to its binocular overlap to allow for triangulation, but if retinal movements would increase resolution binocular triangulation would be feasible[7]. Second, distance estimation could be achieved by moving the eyes across a *binocular ruler*, a term initially coined for lens eyes[8]. One could imagine that two visual neurons, one in the right and one in the left eye, have maximally coinciding receptive fields in space depending on the vergence angles of the eyes[9]. This could allow to triangulate the distance of objects if the vergence angles are known or at least allow the assessment of relative distances. We estimated the range of distances that could be sampled by the innermost ommatidia (assuming a binocular overlap of 5-15deg per eye and an inter-ocular distance of 0.4 mm) to be between 0.8 and 2.3 mm in front of the fly head. This seemingly small range covers roughly the flies' body length and could thus be behaviorally relevant. A connected mechanism has been suggested to underlie hyperacute distance estimation via photocontractions[10].

METHODS

Immunohistochemistry and imaging of retinal muscles and motor neurons
To visualize retinal muscles alongside motor neurons in intact, or semi-intact, fly heads (Fig. 1a-b, Extended Data Movie 3), we expressed mVenus in a split-GAL4 line[11] labeling retinal motor neurons (w+;Mi{Trojan-GAL4DBD.2}VGlut[MI04979-TG4DBD.2]/VT012411-p65ADZp;CsChrimson::mVenus/CsChrimson::mVenus) and performed the following protocol. Flies were anesthetized with CO_2 and briefly washed with 70% ethanol. Heads were isolated. Antennae, maxillary palps and the labrum were removed under 2% paraformaldehyde/PBS/0,1% triton X-100. Heads were fixed in this solution overnight at 4°C. After washing in PBS containing 1% triton X-100, the samples were embedded in 7% agarose and sectioned on Leica Vibratome (VT1000s) either horizontally or coronally, at 0.2 mm. The slices or whole heads were incubated in PBS with 1% triton X-100, 0.5% DMSO and Escin (0.05 mg/ml, Sigma-Aldrich, E1378) containing Texas Red-X Phalloidin (1:50, Life Technologies #T7471), anti-GFP rabbit polyclonal antibodies (1:1000, Thermo Fisher, #A10262) and a chitin-binding dye Calcofluor White (0.1 mg/ml, Sigma-Aldrich #F3543-1G) at room temperature with agitation for two days (slices) or five days (whole mounts). Long incubation times and the presence of surfactants assured better penetration of phalloidin into the muscles. After a series of four ~1hour-long washes in PBS containing surfactants, the sections were incubated for another 24h in the above buffer containing secondary antibodies (1:1000, goat anti-rabbit, Thermo Fisher #A32731). The samples were then washed in PBS/1% triton and fixed for 4 hours in 2% paraformaldehyde to reduce leaching of bound phalloidin from muscles during the subsequent ethanol dehydration step. To avoid artifacts caused by osmotic shrinkage of soft tissue, samples were gradually dehydrated in glycerol (2-80%) and then ethanol (20 to 100 %)[12] and mounted in methyl salicylate for imaging.

Serial optical sections were obtained at 1 µm intervals on a Zeiss 710 confocal microscope with a LD-LCI 25x/0.8 NA objective. Calcofluor White, anti-GFP/anti-rabbit Alexa 488 antibodies and Texas Red phalloidin-treated samples were imaged using a 405, 488 and 594 nm lasers, respectively. Because the blue-green emission of Calcofluor bleeds into the green channel, we applied a sequential scanning approach where we only excited one fluorophore at a time. Images were processed in Fiji (http://fiji.sc/), Icy (http://icy.bioimageanalysis.org/) and Photoshop (Adobe Systems Inc.).

To visual the structures of the orbital ridge (Extended Data Fig. 2) we followed the following protocol: Flies were frozen at -20°C and decapitated in PBS, 0.1% triton X-100. Heads were embedded in 7% agarose and sectioned on Vibratome at 150 µm. The soft tissue was digested by overnight incubation in a mixture of hyaluronidase/trypsin at 0.5 mg/ml in PBS at 37°C. Simultaneously, cuticle was stained with chitin-binding dyes Congo Red (0.25 mg/ml, Sigma-Aldrich #C6767) and Calcofluor White (0.1 mg/ml, Sigma-Aldrich #F3543). The sections were washed, dehydrated and embedded in methyl salicylate. Congo Red and Calcofluor White-treated cuticle was imaged using 405 and 560 nm laser lines, respectively.

Immunohistochemistry and imaging of the L1-L4 and motor neuron split-GAL4 lines in the brain

We dissected the brains out of female flies to visualize neuronal GFP expression a Split-GAL4 line (R34G07-p65.AD;R9B08-GAL4.DBD) that targets L1-L4 neurons[13,14] (Extended Data Fig. 6). Brains were dissected, fixed in 1% PFA (Electron Microscopy Sciences # 15713) in S2 medium (Sigma Aldrich #S0146) and nutated overnight at 4°C. We next blocked specimens in 5% normal goat serum in PBT for 1.5 hours and incubated at least overnight in a primary antibody solution containing 1/30 mouse nc82 (anti-*brp*) (Developmental Studies Hybridoma Bank #nc82-s) and 1/1000 chicken anti-GFP (Rockland #600-901-215). Finally, samples were incubated in a secondary antibody solution containing 1/800 goat anti-chicken-Alexa488 (Life Technologies (Invitrogen) #A11039) and 1/400 goat anti-mouse Alexa594 (Life Technologies (Invitrogen) #A11032), for 3-4 days. Samples were washed in PBT three times after each step. We mounted the brains on slides with the antennal lobe down in the case of L1-L4 and with the antennal lobes up for visualizing the motor neuron. We immersed the brains in VectaShield (Vector Labs), added a coverslip, and took z-stacks with a confocal microscope (Zeiss LSM780) and a Plan-Apochromat 20x/0.8 NA objective. Image processing and analysis have been performed using Fiji software[15].

We checked expression in Kir2.1 flies used for behavior (10XUAS-IVS-Kir2.1-T2A-tdTomato expressed in two split GAL4 lines, Extended Data Fig. 10) using the following steps: One week old flies were anaesthetized on ice and shortly put in a glass staining cup with 80 % ethanol. After 30 sec., flies were put in a staining cup with 1x phosphate buffered saline (PBS). Flies were dissected under the microscope in PBS and brains were stored in a PCR cap with 1:4 solution of 4 % paraformaldehyde (PFA) (Sigma) and 0.1 % phosphate buffered TritonX100 (PBT) until final fixation. Brains were then fixed in 4 % PFA and a drop of 0.1 % PBT for 60 min at room temperature. After fixation, PFA solution was removed, and brains were washed with 0.1 % PBT three times for 20 min at room temperature. The PBT was removed and replaced by 3 % normal goat serum (NGS) in 0.1 % PBT for 30 min at room temperature. The first antibody mix was incubated with Living colors anti-DsRed polyclonal AB (rabbit) (Clontech) (1:200), anti-bruchpilot (nc82) (mouse) (DSHB) (1:200) and 3 % NGS in 0.1 % PBT for 48 h at 4 °C in darkness. Brains were washed with 0.1 % PBT for 5 sec and then three times for 20 min at 4 °C in darkness. Brains were incubated in second antibody mix with Goat anti-rabbit Alexa 568 (Invitrogen) (1:200), Goat anti-mouse Alexa 633 (Invitrogen) (1:200) and 3 % NGS in 0.1 % PBT for 24 h at 4 °C in darkness. Brains were washed with 0.1 % PBT for 5 sec, followed by washing three times for 20 min at 4 °C in darkness. After washing with 0.1 % PBT for 1 h at 4 °C in darkness, brains were mounted on a glass slide with VectaShield. Imaging was carried out at a Leica SP8 confocal microscope with a 20x microscope lens.

Fly genetics and handling

We studied female *Drosophila melanogaster (*or *Drosophila suzukii* for Extended Data Fig. 7), reared in 25℃ incubators with a 12-hour light/dark cycle. In electrophysiology experiments we used flies that were

36-60 hours post-eclosion; in ball-walking and flight experiments we generally used flies 2-5 days post-eclosion. For the tethered and free-behavior gap-crossing experiments in Fig. 5 we used 3-6 day old flies. Flies were raised on standard cornmeal agar media. Unless specified otherwise, we used wild-type Canton-S flies for flight and walking experiments.

All flies used in any behavioral experiments carried at least one copy of the wildtype *white* gene. For silencing of L1-L4 visual neurons we expressed the tetanus toxin light chain (TNT)[16] in R34G07-p65.AD;R9B08-GAL4.DBD and expressed an inactive form of TNT as a control. For electrophysiological recordings and optogenetic activation (Figure 2), we used flies with the following genotype: w+;R39H04-lexA/ lexAop myrGFP;R44A07-GAL4/20xUAS-CsChrimson::mVenus. Flies again were raised on retinal-poor food (standard cornmeal media) and transferred to retinal enriched food after eclosion (0.4 mM all trans-Retinal, CAS# 116-31-4, added to our standard cornmeal food). Flies were kept in light-shielded boxes to in darkened boxes to minimize CsChrimson activation during development and as adults. For optogenetic activation in Extended Data Fig. 2 we were driving 20xUAS-CsChrimson::mVenus in R44A07-AD;R13D09-DBD. To guide recordings in Extended Data Fig. 2, we drove mCD8GFP in VT037804-GAL4. In Extended Data Fig. 8, we targeted horizontal system (HS) and vertical system (VS) cells by driving green fluorescent protein (eGFP) in two GAL4 lines: w+;UAS-2xeGFP/+;GMR81G07-GAL4 and w+;UAS-2xeGFP/tshirt-gal80; VT58487-Gal4/+.

To impair retinal movements (Fig. 5, Extended Data Fig. 10 we expressed a mouse Kir2.1 ion channel (w+/w-;10XUAS-IVS-Kir2.1-T2A-tdTomato, tubP-Gal80[ts]) in retinal motor neurons or a mutated Kir2.1 channel, which is non-conducting, as a control (w+/w-;10XUAS-IVS-Kir2.1Mut-T2A-tdTomato; tubP-GAL80[ts]). We used two split-GAL4 driver lines for retinal motor neurons, which we generated: R44A07-AD;R13D09-DBD and R14B04-AD;R13D09-DBD.

Flies were anesthetized on a Peltier stage at ~4℃ and were glued to a custom stage[17] for electrophysiological experiments (Fig. 2, Extended Data Figs. 2,8) or when we needed to image photoreceptor movements with a water immersion objective (Fig. 1f). We used a blue-light activated glue (Bondic, Canada). For behavioral flight experiments, we also anesthetized flies on a stage at ~4°C and then we glued them to a tungsten pin. In all experiments (except the free behavior in Fig. 5), the head was rigidly tethered to the thorax, or to the fly holder. For electrophysiology experiments we additionally glued the proboscis in place.

Recording the movements of the deep pseudopupil and photoreceptor tips directly

We imaged the deep pseudopupil with multiple different air objective/lens and camera combinations (see below). We used the software package *fview*[18] to capture movies of the deep pseudopupil for either off-line tracking or in combination with custom-written real-time tracking software (see below).
For the simultaneous imaging of the deep pseudopupil via an air lens and of photoreceptors, directly, via a water immersion objective (Fig. 1f), we used two externally triggered Chameleon 3 cameras (CM3-U3-13Y3M, FLIR) one fitted with a 4x Infinistix air lens (Infinity Photo-Optical) and the other camera recording the photoreceptors through a 40x/0.8 NA (Olympus) water-dipping microscope objective. The fly head was glued onto a custom-made holder designed for electrophysiological recordings in flight[17]. The fly head was tilted in such a manner as to make the dorsal part of the eye visible in the 40x water-dipping image while the ventral part of the eye was visible to the 4x air lens. The fly performed tethered flight during these simultaneous measurements, but we did not measure its steering behavior.

For Fig. 1d, where we measured photoreceptor movements triggered by optogenetic activation of motor neurons, we used a CoolSnap EZ camera (Photometrics) visualizing the photoreceptors through a 40x/0.8 NA (Olympus) water-dipping objective. Using a 90:10 beamsplitter (Thorlabs, BSX10R) we sent focused red light (660 nm, Thorlabs M660L4-C1) down on the fly head, to activate CsChrimson, via the epi-fluorescence port of the microscope, while visualizing the position of the photoreceptors in the camera. In this experiment the fly was tethered to a metal pin and fully immersed in water so as to allow us to rotate the fly under the microscope and obtain the highest possible quality images of the photoreceptors around the midline of the eye. When we performed our initial optogenetic activation of the muscles in the context of imaging the deep pseudopupil (Fig. 1e), we used a Chameleon 3 (CM3-U3-13Y3M, FLIR) camera with a 6x Infinistix lenses (Infinity Photo-Optical). We adjusted the red-light intensity in these experiments upward until the photoreceptor or deep pseudopupil excursions stopped growing in size.

To measure deep pseudopupil and flight steering responses to visual stimuli (Fig. 3), we used two Prosilica GE680 cameras (Allied Vision Technologies), one for each eye, each imaging with a 6x Infinistix lens (Infinity Photo-Optical). To illuminate the pseudopupil, we placed an 850 nm fiber optic abutting the fly's head from behind. To get crisp images of the pseudopupil it was crucial to move the lightguide as close as possible to the fly head so as to maximize light that scattered in the head capsule and out of the eyes in comparison to stray light hitting the eyes from the outside. Synchronized movies from the two cameras were recorded at 100 Hz and the movement of the deep pseudopupil was tracked offline using a custom written algorithm (see below).

In electrophysiology experiments in Figure 2 we recorded both retinas using a Chameleon 3 cameras and InfiniStix lenses (3x, 44mm, Infinity Photo-Optical) at 60 Hz. To activate the motoneurons expressing ChrimsonCS, we used an achromatic lens pair (Thorlabs MAP10100100-A) to focus a dot of red light between the eyes (660 nm LED, Thorlabs M660FPI, light guide: M15L01). We filtered this wavelength using bandpass filters in the cameras (725/ 50nm, Edmund Optics #86-943). To For the HS/VS recordings in Extended Data Fig. 8, we collected movies of the left-eye's deep pseudopupil at 200 Hz using a Prosilica GE680 camera and a 4x InfiniStix lens. We illuminated the deep pseudopupil in these experiments by shining 735 nm light (Thorlabs M735L3-C1) focused onto the head through the 40x water-dipping objective.

In ball-walking experiments (Extended Data Fig. 7), we measured retinal movements and walking behavior by placing flies on a small airborne ball (see below) and using two Chameleon 3 (CM3-U3-13Y3M, FLIR) cameras with 6x Infinistix lenses (Infinity Photo-Optical) to visualize the deep pseudopupils of each eye. The light guide (200 µm, Thorlabs M87L01) used to illuminate the deep pseudopupil was placed slightly above and behind the head. It was connected to an 850 nm LED (Thorlabs M850F2).

In tethered gap-crossing experiments (Fig. 5 and Extended Data Fig. 9d), we visualized the movements of the deep pseudopupil with 6x Infinistix lenses (Infinity Photo-Optical), one per eye. In Extended Data Fig. 9a-c, we only tracked the pseudopupil of the right eye. We illuminated the head with an 850 nm LED (Thorlabs M850F2) coupled to a light guide (105 µm, Thorlabs M15L01) that delivered light to a matched achromatic pair of lenses (MAP10100100-A, Thorlabs), which focused the infrared light to a small spot on the head from slightly above and behind the fly.

Flight and walking behavior on a ball or wheel

In experiments with tethered, flying flies (Fig. 3), we measured the right and left wing stroke amplitudes using a wingbeat analyzer (JFI Electronics Laboratory, University of Chicago, Chicago, IL)[19] and took the difference between these two signals as the fly's steering response. Specifically, flies were illuminated with an infrared diode (880 nm wavelength) from above, so that the shadows of the flapping wings can be recorded with two photodiodes placed below the flies. The signal from the photodiodes oscillates in synchrony with the wings flapping forward and backward, with the maximum (inverted) photodiode signal on each cycle indicating, quantitatively, the maximum forward wingstroke angle achieved on that stroke, otherwise known as the wingbeat amplitude (WBA). The difference between the left and right wingbeat amplitude (L–R WBA) is correlated with yaw torque[20] and thus provides a good measure of the direction and strength in which the fly is attempting to steer. We used a second LED at a slightly different wavelength (850 nm), to illuminate the fly head for visualizing the deep pseudopupil. We placed a bandpass filter (880 ±5 nm, Edmund Optics, NJ, NT43-150) over the photodiodes that record the wing movements to prevent any impact of the 850 nm light on wing tracking. We recorded the left and right wingbeat amplitudes, camera triggers, and voltages encoding the visual stimuli on our panoramic LED display at 1 kHz using a Digidata 1440a (Molecular Devices, CA). At the beginning and end of each experiment we paused the camera triggers and used these pauses to precisely align the pseudopupil estimates on each frame to the L–R WBA and visual-stimulus voltage signals that were simultaneously recorded.

We developed a tethered gap-crossing paradigm (Fig. 5a-e and Extended Data Fig. 9). We 3D printed fly-sized wheels with a Projet MJP 3600 series 3D printer (30µm resolution) using VisiJet M3 Crystal material. We press-fit a precision shaft (Swissjewel, N-1D) through the middle of the wheel and placed that shaft between two spring-loaded jewel bearings (Swissjewel, VS-30), which allowed the wheel to rotate with low friction. The wheels had a 5 mm wide walking surface and a radius of 4 mm. Each wheel weighed ~89 mg, with a rotational inertia around the central axis of 0.87 gmm^2. The wheel had two equally sized gaps, 180˚ apart. We spray painted the wheel black and we painted white, horizontal stripes on the walls of one gap and white, vertical stripes on the walls of the other gap. We 3D printed the stripes to be slightly protruding, which allowed us to paint accurate stripes with a fine-point sharpie. The vertical stripes had a 1.4 mm wavelength (0.7 mm white, 0.7 mm dark) and the horizontal stripes had a 1 mm wavelength (0.5 mm white and 0.5 mm dark). Both these periodicities corresponded to a gratings with an optical wavelength of ~20˚ for a fly standing on the far edge of the gap. Our algorithm for analyzing the gap-crossing wheel data is described later in the Methods (see below).

In experiments with tethered, flies walking on a spherical ball (Extended Data Figs. 7), we tracked the flies ball-walking behavior as reported previously[21]. Specifically, the flies walked on a 6.35 mm (1/4 inch) diameter ball carved by hand from Last-A-Foam FR-4618 (General Plastics) using a custom machined steel hemispherical cutter[22]. The ball was placed at the top of an aluminum post with a 6.75 mm (17/64 inch) diameter hemispherical cavity machined into the top[23]. Air was flowed into a 1 mm hole at the bottom of the hemispherical cavity at just enough of a rate to support the ball floating freely. The ball was marked with irregular black spots and tracked using FicTrac, which uses a map of the ball's black-spot pattern to calculate its 3D angular position on each frame [24]. The software we used to display visual stimuli (see below) and FicTrac ran on separate computers—one Windows machine and one Linux machine—and we therefore used network time protocol (NTP) to synchronize the system clocks between the two computers to below 2 ms. In this way, the timestamps from the ball-position and visual-stimulus

data files could be accurately compared. The Linux computer was set up as the NTP server and the Windows computer was configured to set its system clock from this server over the local network.

Visual Stimuli

For experiments in tethered, flying or quiescent flies (Fig. 3 and 4a-c), we presented visual stimuli via a cylindrical green LED (570 nm) visual display that extended 360° in azimuth and 45° in elevation (IORodeo, CA)[25]. We removed LEDs covering 30° of azimuthal space centered around 60° to the fly's left and 60° to the fly's right, which allowed us to bring two cameras close to the fly's head, one for imaging the deep pseudopupil of the left eye and the other for imaging the deep pseudopupil of the right eye (Fig. 3a). Each LED pixel subtended 1.875° around the yaw axis. In one set of experiments (Fig. 3), we presented vertical gratings (15° wavelength) that moved with a temporal frequency of 1 Hz. The gratings were visible either panoramically or just in one hemisphere (with mean luminance on the other side). We also presented expansion stimuli (where the gratings moved front-to-back on both sides) or contraction stimuli (where the gratings moved back-to-front on both sides) as well as a horizontal grating that moved up or down. For all stimuli aside from the panoramic gratings, the frontal ±30° of the arena was kept at mean luminance (i.e., without a moving stimulus) so as to ensure no motion stimulation of the contralateral eye. We used green-scale dithering via three (vertical grating) or two (horizontal grating) intensity levels for each LED pixel to allow for edges in visual patterns to appear to move more smoothly across frames. Trials were 5 s long and each stimulus was presented with two motion directions (left/right or up/down), repeating all the stimuli ten times for each fly. Retinal movements in response to grating motion were recorded in the same cohort of 10 flies in both non-flight and during tethered flight. For some conditions only eight or nine flies had enough valid trials in tethered flight. Using the same experimental setup, we also presented stationary visual environments and recorded eye movements in quiescent and flying flies that occur spontaneously (i.e., with no moving stimulus to elicit them) (Fig. 4a-c). Each stationary panorama—dark screen, bright screen, vertical grating, horizontal grating—was presented five times for 30 s each time. In electrophysiological experiments, we used a cylindrical green LED display that covered 216° in azimuth and 76° in elevation (IORodeo, CA)[25]. For the experiments in in Extended Data Fig. 8, we presented the same stationary stimuli just described for 15 s—dark screen, bright screen, vertical grating, horizontal grating—as well as horizontal and vertical grating motion (1 Hz temporal frequency, for 5 s) to assess the motion-direction preferences of the recorded cells.

In tethered, ball-walking flies (Extended Data Figures 7,10,11) we presented visual stimuli with a projector that allowed us to accurately present gratings with a significantly smaller wavelength (as small as 1°) than those we could present on our LED display. Specifically, visual stimuli were displayed with a TI DLP LightCrafter 4500 projector, reflected off of a flat mirror (https://firstsurfacemirror.com), onto a conical screen made out of white card stock supported by an acrylic frame. The screen covered 360° in azimuth and 90° in elevation, with ~20° of the screen blocked from view, directly behind the fly, by a camera apparatus used to track the ball. Visual stimuli were generated using ViRMEn, an open source, Matlab-based virtual reality platform[26]. We created virtual striped cylinders in the ViRMEn virtual world and presented these to the fly. To measure the flies' spatial acuity (Extended Data Fig. 7), we created virtual cylinders textured with a repeating sinusoidal grating of the desired spatial wavelength. The cylinder gently faded to grey at the top and bottom of the conical screen, along a gaussian profile, so as to minimize the presence of sharp edges. We then rotated this virtual cylinder around the fly, and we measured the resulting retinal optokinetic responses and walking optomotor responses.

Electrophysiology

We performed whole-cell current-clamp measurements from LC14 cells (i.e., dorsal cluster neurons) (Fig. 2 and Extended Data Fig. 2) and from HS and VS cells (Extended Data Fig. 8), using methods described previously[6,27]. We recorded from LC14 cells on the right side of the brain and tracked the retinas of both eyes during those experiments; we recorded from HS/VS cells on the left side of the brain and tracked the left retina only during those experiments. Specifically, we affixed flies to a custom stage that allow for patch-clamp recording in tethered, flying flies[17]. We dissected the cuticle, muscles and trachea above the brain and perfused the tissue with extracellular solution (275-285 mOSM) that contained in mM: 103 NaCl, 3 KCl, 5 N-Tris(hydroxymethyl) methyl-2-aminoethanesulfonic acid (TES), 10 Trehalose, 10 Glucose, 2 Sucrose, 26 NaHCO3, 1 NaH2PO4, 1.5 CaCl2, 4 MgCl2. We bubbled this solution with 95% O2/5% CO2 to achieve a pH of 7.3. As described previously[17], we used locally applied collagenese IV in extracellular solution (at a maximum concentration of 0.5 mg/mL) and mechanical pressure to break through the neurilemma and perineurial glial sheath, providing access to the HS and VS cell bodies. For recordings from LC14 cells (Fig. 2) we used forceps to remove the neurilemma and perineurial glia, and did not use collagenase IV. Pipettes were filled with intracellular solution that contained in mM: 140 K-Aspartate, 1 KCl, 10 HEPES, 1 EGTA, 0.5 Na3GTP, and 4 MgATP, 0.02 Alexa-568- hydrazide-Na and 13 Biocytin hydrazide. The membrane voltage was amplified (MultiClamp 700B, Molecular Devices), digitized at 10 kHz (Digidata 1440a, Molecular Devices), and saved to a computer (pClamp software suite, Molecular Devices). Voltage measurements have been corrected for a 13 mV junction potential. Throughout data collection, the extracellular bath was held at 25-26℃, slightly higher than room temperature, because this temperature seemed to promote an increased rate of retinal movements. Pipette resistances ranged from 4.5 to 8 MΩ.

Tracking the deep pseudopupil

We tracked the deep pseudopupil using a custom algorithm written in python 2.7 using openCV (https://opencv.org). We defined a region of interest in the image that included all potential pseudopupil positions but excluded other bright cuticular features, mainly near the edge of the eye. We smoothed the image to minimize pixel noise, sometimes enhanced local contrast, binarized the image, and subsequently calculated the contours of the resultant blob, saving the centroid position of this blob as the x-y position of the deep pseudopupil in pixel coordinates. The parameters for filtering, contrast enhancement and thresholding were adjusted for each eye and experiment because the exact nature of the illumination changed between flies. All parameters, however, were held constant for all trials for a given experiment. We tracked the deep pseudopupil movements either offline, or in later experiments, online.

To convert how many pixels the pseudopupil moved on the camera image to an estimate of how many degrees of visual space the photoreceptors field-of-view moved, we performed the following transformation with Fiji (https://imagej.net/Fiji). Using a single image frame of each fly eye, we measured the distance in pixels between the midpoints of the outermost photoreceptors (with one photoreceptor between them). Assuming that *D. melanogaster* have an inter-ommatidial angle of ~5° [3,28], this distance should reflect approximately 10° of angular space. For the conversion of *Drosophila suzukii* retinal movements from pixel units to degrees, we assumed an inter-ommatidial angle of 3.25° based on the sign inversion of the responses at a wavelength of approximately 6.5° (Extended Data Fig. 7d). In ball- and wheel-walking experiments, we used a similar algorithm as described above (choice of a region of interest, filtering, thresholding), but we only saved the x-y center position of the pseudopupil, with a timestamp, to our data file, instead of saving the entire movie and tracking offline. We measured the size

of the pseudopupils in pixels in single frames as described above and calibrated the pixel movements with an averaged pseudopupil size.

Analyzing shifts in receptive fields due to retinal movements

In Fig. 2 we recorded from six LC14 neurons, while optogenetically activating retinal motoneurons. We tracked both retinas but only analyzed the movement of the right retina as we were recording in the right visual hemisphere. The cameras viewed the deep pseudopupil from a rear, downward position (due to spatial constraints) and the cameras were in this case not aligned to the visual stimulus. In order to estimate the horizontal shifts of the deep pseudopupil relative to visual stimulus, we inclined the screen to the head of the fly to align so that the vertical bars on the screen move orthogonal to the h-row of the ommatidial array (Fig. 4 and Fig. S3 in ref. 6). We then rotated the tracking vectors to obtain the deep pseudopupil movements along the h-row. Each fly was shown several repetitions of a vertical bar moving clockwise and counterclockwise, both with and without optogenetic lights on. Rarely, we observed unexplained depolarizations that recovered after several seconds, and we thus removed trials in which the *Vm* was more than 10 mV higher than the overall mean *Vm* (2 out of 170 total trials across six flies, note that both excluded trials were from one fly). We then calculated the average, baseline-subtracted membrane voltage for each fly across all trials for both motion directions with and without optogenetic activation. To compare these single fly averages across the population we shifted the curves to align the peaks of the receptive fields for no optogenetic activation. The peak was determined for lowpass filtered data (Gaussian filter, s = 10ms), but the unfiltered curves are plotted. This allowed us to calculate mean receptive fields across the population despite slightly varying peak response in yaw. We calculated the mean angular shift of the right deep pseudopupil for each fly while the stripe was in the ipsilateral half of the screen, including a 25˚ wedge into the contralateral hemifield; this region was certain to any LC14 receptive field we recorded. Finally, we normalized the receptive fields of each fly to their peak (see above), and shifted the mean receptive fields by the mean angular separation of the deep pseudopupils in the two conditions.

Analyzing optokinetic responses in flight and quiescence

In plots showing the population responses of flying or quiescent flies to visual stimuli (Figs. 3 and Extended Data Fig. 5) we calculated the mean for each fly over all valid stimuli presentations, with invalid trials being those in which the retina was poorly tracked, or, in flight conditions, when the fly stopped flying. The right and left wingbeat amplitude signals were lowpass filtered using a butterworth filter (order=8; cutoff frequency=10 Hz) applied bidirectionally to not introduce a time shift. All traces in Figures 3 and Extended Data Fig. 5 were baseline corrected by subtracting the mean signal in a 1.5 s window before the onset of visual motion from the entire trace. We convolved the retinal position trace with a Gaussian filter (s=4ms) and then took its derivative. In this filtered velocity trace, we isolated retinal saccades by finding local minima and maxima whose magnitude exceeded two standard deviations from the mean velocity during that trial (Extended Data Fig. 3). In Extended Data Fig. 3, we triggered on saccades thus detected in the right retina and plotted concomitant movements of the left retina, as well as the L–R WBA wing steering signal, when flies were flying. For the amplitude versus velocity plots (Extended Data Fig. 4) we estimated the amplitude of isolated saccades by subtracting the maximum and minimum retinal position observed in a 200 ms time window surrounding the peak velocity. We used the peak velocity in the same time window for the saccade's velocity. We estimated the initial speed of retinal responses from the mean over all trials for each fly in a 100 ms time window starting 50 ms after the onset of visual motion (Extended Data Fig. 5) for three conditions (full-field rightward, rightward motion in the right hemisphere and bilateral front-to-back motion) in flight and quiescence.

For Extended Data Fig. 6, we calculated an optomotor index as a measure of how strongly flies respond to visual motion. This index was calculated by subtracting the mean response strength during visual motion in both motion directions from the mean signal in a 1.5 s window before the onset of visual motion, and then subtracting these two values from each other. To test whether the difference in optomotor index of silenced and control flies differed was significant we used a two-sided t-test for the means of two independent samples. The differences in optomotor index for retinal movements between the two genotypes in non-flight and flight was significant with $p<0.0001$ in both cases. The difference in the optomotor index of the wing movements was also significant ($p < 0.00001$).

Analyzing retinal movements in stationary environments

In Figure 4, to select saccades we found local maxima in the retinal velocity trace (filtered as above) that exceeded three standard deviations of the mean on that trial. We show smoothed saccades (Gaussian filter, s.d.=4ms) from 10 flies triggered on retinal movements in the right eye.

Analyzing electrophysiological responses to spontaneous retinal movements

In Extended Data Fig. 8, to test whether HS and VS cells activate with eye movements, we simultaneously recorded their membrane voltage (*Vm*) alongside the fly's retinal movements in the context of varying stationary scenes, presented for 15 s each (see above). At the end of each recording, we also measured the *Vm* response of the cell to moving gratings (1 Hz temporal frequency), presented for multiple 5 s presentations, in both the preferred direction (leftward for HS cells and downward for VS cells) and in the opposite, or null, direction (e.g., Extended Data Fig. 8a). We recorded from seven VS cells that responded best to vertical motion and five HS cells, which respond best to horizontal motion. In each experiment we presented a uniformly lit arena, darkness, a stationary vertical and a stationary horizontal grating (both at 15° wavelength). We placed a shroud over the rig, such that when the visual display was turned off (darkness), there should have been very little light reaching the fly's eyes. For VS cells, we focused our analysis on vertical retinal movements and for HS cells we focused on horizontal movements (because movements along the other axes are expected to create visual motion sensed less effectively by the cells). Retinal position traces were filtered using a butterworth filter (order=8, cutoff frequency=100 Hz) applied bidirectionally. We selected upward/downward retinal movements for VS cells and rightward/leftward retinal movements for HS cells, by finding local maxima or minima of the derivative of the smoothed (Gaussian, s=50 ms) retinal position trace. We analyzed those retinal movements, i.e., likely saccades, in which the retinal position change (without filtering) exceeded 0.15° in a window from 200-ms before to 300-ms after the local minimum or maximum was found. We used this requirement rather than the minimum deviation from mean used for the behavioral data, since the retinal movements varied largely from fly to fly during electrophysiological recordings, and we wished to compare retinal movements of a similar amplitude across individuals. We did not analyze the first 1.5 s after the onset of a stationary stimulus to avoid any stimulus-flash-driven saccades or flash *Vm* responses from the neuron. The number of saccades per fly varied depending on the quality of the preparation and was typically 10-50 total saccades in each direction per fly and visual condition, with the smallest number of saccades being 9 for one fly in the grating condition. For each fly and stimulus condition, we calculated the average pseudopupil and *Vm* time course and the calculated the pooled time course of all cells (N=12) for a darkness, a uniformly lit arena, and the stationary grating orientation with bars oriented orthogonally to the preferred/null axis of the cell (i.e., vertical gratings for HS cells and horizontal gratings for VS cells). We tested whether the small *Vm* fluctuation observed in darkness was significant by comparing single fly *Vm* averages in a 20 ms window around the onset of a saccade compared to the averages in a 50

ms window starting 100 ms before the saccade. The differences between those two values were significant for both eye movement directions (two-sided Wilcoxon signed rank test, statistic=6.0, p=0.0096 for downward/leftward retinal movements and statistic=3.0, p=0.005 for upward/rightward movements). To test for significance of the visual responses we compared the averages in a 50 ms window around the peak response to the average in a 50 ms window at the onset of the saccade. The differences were significant for both retinal movement directions (two-sided Wilcoxon signed rank test, statistic=7.0, p=0.01, for preferred movements, and statistic=11.0, p=0.028 for null direction responses).

Analyzing tethered gap-crossing data

To track the gap-crossing wheel's angle over time, we added two, prominent, 3D-printed marks (one diamond and one line) to the mid-point of the wheel's spokes and we painted these markers white. We trained a DeepLabCut (version 2.2.0.6) model, with default parameters, on 160 frames where we labeled the XY position of these two markers, from 8 videos for a total of 1,030,000 iterations, yielding a test error of 8.64 pixels (image size 1280 x 1024 pixels). The mature neural network could take in images of the wheel and output the XY position of the markers. We wrote an algorithm in Python that used the XY positions of these two markers to estimate the wheel's angle on each frame. On any given frame, one marker (the diamond or the line) could be occluded by the base that held the jewel bearings; however, at least one marker was always visible, making the positional tracking of the wheel reliable, as determined by visual inspection of many movies. We tracked the wheel at 150 Hz using a Chameleon 3 (CM3-U3-13Y3M, FLIR) with a Computar Macro Zoom Lens (COMLM3XMP, B&H Photo).

We imaged, at 70-100 Hz, the left and right pseudopupils, each with a Chameleon 3 (CM3-U3-13Y3M, FLIR) affixed with a 6x Infinistix lens (118600, Infinity Photo-Optical). After data acquisition, we created a common 100 Hz time base to which we placed the pseudopupil and wheel data, using linear interpolation as needed. We isolated moments when the fly crossed a gap in the forward direction by defining two angular thresholds, one for each gap, and requiring the wheel to have rotated across the relevant threshold. To define a genuine gap crossing event, we also required that the mean's wheel position stay > 0.1 rad (~5.7°) above this threshold in the two seconds after gap crossing, so as to avoid annotating unsuccessful lunges or reaches as crossing events, that is, where the fly quickly returns to the its pre-crossing location after crossing threshold briefly.

Analyzing free-behavior gap-crossing data

To test for the ability of flies to cross gaps in free behavior (Extended Data Fig. 10), we used a 3D-printed *catwalk* (40 mm long, 10 mm high and 5 mm wide) made of black LCD resin, which had a 3.5-mm long and 7-mm deep gap in the middle. We placed the catwalk on a circular black acrylic platform, which was 50 mm in diameter and surrounded by water (to prevent escape). The platform was also surrounded by a white cardboard cylinder to provide a uniform visual background. The arena was evenly illuminated by two overhead white LED lights (HETP, OO7). We cut a hole in the cardboard to allow us to film the catwalk from the side at 50 Hz (camera BFS-U3-16S2M-CS, Teledyne FLIR, Oregon, U.S.; lens Fujinon HF12XA-5M, FUJIFILM, Tokyo, Japan). At least 24 h before each trial, we cut the flies' wings under CO2-induced anesthesia. We also starved flies of food, but not water, for at least 4 h before a trial. We alternated running experimental and control flies in consecutive trials. In each trial, we placed a single fly on the circular platform and after her first voluntary visit to the catwalk, the fly was given 10 min. to freely explore. We used DeepLabCut (version 2.2.0.6) to track the position of the fly's abdominal tip as she traversed the gap. Prior to video analysis, the DeepLabCut model was trained, with default parameters, on 700 labeled frames from 28 videos for a total of 350000 iterations, yielding a test error of

2.54 pixels (image size 1440 x 430 pixels). We used this model to analyze all experimental videos. We tested 36-37 female flies per genotype. To analyze the behavior, we determined when the abdomen tip of a fly crossed the midline of a gap. We then determined, in a 400 ms window starting 200 ms before the midline crossing moment, the minimum position of the abdomen tip, as a proxy of the height at which the fly crossed the gap. We then calculated the median of these crossing heights of all crossing events for single flies and compared these distributions across genotypes using a Welch test.

Supplementary references

1. Juusola, M. *et al.* Microsaccadic sampling of moving image information provides Drosophila hyperacute vision. *Elife* **6**, e26117 (2017).
2. Keesey, I. W. *et al.* Inverse resource allocation between vision and olfaction across the genus Drosophila. *Nature Communications* **10**, 3–22 (2019).
3. Götz, K. G. Die optischen Übertragungseigenschaften der Komplexaugen von Drosophila. *Biological Cybernetics* **2**, 215–221 (1965).
4. Salem, W., Cellini, B., Frye, M. A. & Mongeau, J.-M. Fly eyes are not still: a motion illusion in Drosophila flight supports parallel visual processing. *J Exp Biol* **223**, jeb212316 (2020).
5. Schnell, B. *et al.* Processing of horizontal optic flow in three visual interneurons of the Drosophila brain. *Journal of Neurophysiology* **103**, 1646–1657 (2010).
6. Kim, A. J., Fenk, L. M., Lyu, C. & Maimon, G. Quantitative predictions orchestrate visual signaling in Drosophila. *Cell* **168**, 280–294 (2017).
7. Viollet, S. Vibrating Makes for Better Seeing: From the Fly's Micro-Eye Movements to Hyperacute Visual Sensors. *Front Bioeng Biotechnol* **2**, 9 (2014).
8. Collett, T. S. & Harkness, L. I. K. Depth vision in animals. in *Analysis of visual behaviour* (eds. Ingle, D. J., Goodale, M. A. & Mansfield, R. J. W.) 111–176 (Cambridge, MA: MIT Press, 1982).
9. Hengstenberg, R. Das Augenmuskelsystem der Stubenfliege Musca domestica. *Kybernetik* **9**, 56–77 (1971).
10. Joni, K. *et al.* Binocular mirror–symmetric microsaccadic sampling enables Drosophila hyperacute 3D vision. *Proceedings of the National Academy of Sciences* **119**, e2109717119 (2022).
11. Pfeiffer, B. D. *et al.* Refinement of Tools for Targeted Gene Expression in Drosophila. *Genetics* **186**, 735 LP – 755 (2010).
12. Ott, S. R. Confocal microscopy in large insect brains: Zinc–formaldehyde fixation improves synapsin immunostaining and preservation of morphology in whole-mounts. *Journal of Neuroscience Methods* **172**, 220–230 (2008).
13. Strother, J. A., Nern, A. & Reiser, M. B. Direct observation of on and off pathways in the Drosophila visual system. *Current Biology* **24**, 976–983 (2014).
14. Tuthill, J. C., Nern, A., Holtz, S. L., Rubin, G. M. & Reiser, M. B. Contributions of the 12 neuron classes in the fly lamina to motion vision. *Neuron* **79**, 128–140 (2013).
15. Schindelin, J. *et al.* Fiji: an open-source platform for biological-image analysis. *Nat Methods* **9**, 676–682 (2012).
16. Sweeney, S. T., Broadie, K., Keane, J., Niemann, H. & Kane, C. J. O. Targeted expression of tetanus toxin light chain in Drosophila specifically eliminates synaptic transmission and causes behavioral defects. *Neuron* **14**, 341–351 (1995).
17. Maimon, G., Straw, A. D. & Dickinson, M. H. Active flight increases the gain of visual motion processing in Drosophila. *Nature Neuroscience* **13**, 393–399 (2010).
18. Straw, A. D. & Dickinson, M. H. Motmot, an open-source toolkit for realtime video acquisition and analysis. *Source Code for Biology and Medicine* **4**, 5 (2009).
19. Götz, K. G. Course-Control, Metabolism and Wing Interference During Ultralong Tethered Flight in Drosophila Melanogaster. *Journal of Experimental Biology* **128**, 35–46 (1987).

20. Tammero, L. F., Frye, M. A. & Dickinson, M. H. Spatial organization of visuomotor reflexes in Drosophila. *Journal of Experimental Biology* **207**, 113–122 (2004).
21. Green, J. *et al.* A neural circuit architecture for angular integration in Drosophila. *Nature* **546**, 101–106 (2017).
22. Seelig, J. D. *et al.* Two-photon calcium imaging from head-fixed Drosophila during optomotor walking behavior. *Nature Methods* **7**, 535–540 (2010).
23. Seelig, J. D. & Jayaraman, V. Neural dynamics for landmark orientation and angular path integration. *Nature* **521**, 186 (2015).
24. Moore, R. J. D. *et al.* FicTrac: A visual method for tracking spherical motion and generating fictive animal paths. *Journal of Neuroscience Methods* **225**, 106–119 (2014).
25. Reiser, M. B. & Dickinson, M. H. A modular display system for insect behavioral neuroscience. *Journal of Neuroscience Methods* **167**, 127–139 (2008).
26. Aronov, D. & Tank, D. W. Engagement of Neural Circuits Underlying 2D Spatial Navigation in a Rodent Virtual Reality System. *Neuron* **84**, 442–456 (2014).
27. Kim, A. J., Fitzgerald, J. K. & Maimon, G. Cellular evidence for efference copy in Drosophila visuomotor processing. *Nature Neuroscience* **18**, 1247–1255 (2015).
28. Stavenga, D. G. Angular and spectral sensitivity of fly photoreceptors. II. Dependence on facet lens F-number and rhabdomere type in Drosophila. *Journal of Comparative Physiology A: Neuroethology, Sensory, Neural, and Behavioral Physiology* **189**, 189–202 (2003).
